# Water-Stable Carborane-Based Eu^3+^/Tb^3+^ Metal–Organic Frameworks for Tunable Time-Dependent Emission Color and Their Application in Anticounterfeiting Bar-Coding

**DOI:** 10.1021/acs.chemmater.2c00323

**Published:** 2022-04-29

**Authors:** Zhen Li, Rosario Núñez, Mark E. Light, Eliseo Ruiz, Francesc Teixidor, Clara Viñas, Daniel Ruiz-Molina, Claudio Roscini, José Giner Planas

**Affiliations:** † Institut de Ciència de Materials de Barcelona (ICMAB-CSIC), Campus UAB, 08193 Bellaterra, Spain; ‡ Department of Chemistry, University of Southampton, Highfield, Southampton SO17 1BJ, U.K.; § Departament de Química Inorgànica i Orgànica and Institut de Recerca de Química Teòrica i Computacional, Universitat de Barcelona, Diagonal 645, 08028 Barcelona, Spain; ∥ Catalan Institute of Nanoscience and Nanotechnology (ICN2), CSIC, and The Barcelona Institute of Science and Technology (BIST), Campus UAB, Bellaterra, Barcelona 08193, Spain

## Abstract

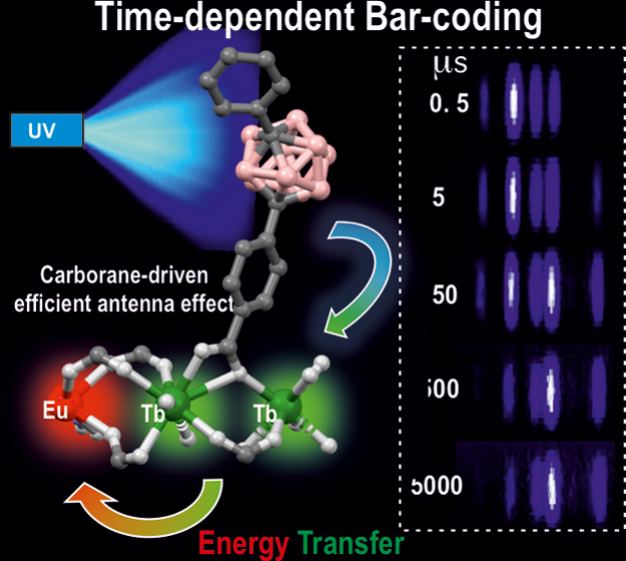

Luminescent lanthanide metal–organic frameworks (Ln-MOFs) have been shown to exhibit relevant optical properties of interest for practical applications, though their implementation still remains a challenge. To be suitable for practical applications, Ln-MOFs must be not only water stable but also printable, easy to prepare, and produced in high yields. Herein, we design and synthesize a series of ***m*****CB-Eu***_**y**_***Tb**_**1–*y***_ (*y* = 0–1) MOFs using a highly hydrophobic ligand *m*CBL1: 1,7-di(4-carboxyphenyl)-1,7-dicarba-*closo*-dodecaborane. The new materials are stable in water and at high temperature. Tunable emission from green to red, energy transfer (ET) from Tb^3+^ to Eu^3+^, and time-dependent emission of the series of mixed-metal ***m*****CB-Eu***_**y**_***Tb**_**1–*y***_ MOFs are reported. An outstanding increase in the quantum yield (QY) of 239% of *m*CB-Eu (20.5%) in the mixed ***m*CB-Eu_0.1_Tb_0.9_** (69.2%) is achieved, along with an increased and tunable lifetime luminescence (from about 0.5 to 10 000 μs), all of these promoted by a highly effective ET process. The observed time-dependent emission (and color), in addition to the high QY, provides a simple method for designing high-security anticounterfeiting materials. We report a convenient method to prepare mixed-metal Eu/Tb coordination polymers (CPs) that are printable from water inks for potential applications, among which anticounterfeiting and bar-coding have been selected as a proof-of-concept.

## Introduction

Porous coordination polymers (CPs), also known as metal–organic frameworks (MOFs), are a class of highly crystalline materials formed by metal ions or metal clusters connected by multitopic organic linkers, which have attracted extensive attention over the past few decades.^1−4^ Their large surface areas, framework flexibility, and tunable pore surface properties, as well as “tailor-made” framework functionalities, empower them to be promising candidates for a diverse range of applications.^2,5−13^ Especially interesting is the combination of MOFs with lanthanide (Ln) ions resulting in inherent optical properties, including high luminescence quantum yields, narrow and strong emission bands, large Stokes shifts, long luminescence lifetimes, and an emission wavelength undisturbed by the surrounding chemical environment.^14,15^ Their luminescence is associated with an energy transfer (ET) from the ligand, acting as an antenna, owing to its larger extinction coefficient, to the accepting electronic levels of the emitting lanthanides and it is potentially interesting in a variety of applications, such as e.g., sensors, optoelectronic and in solid-state lighting (SSL) devices, or bioimaging among others.^16−22^ Of particular interest would be the exploitation of emissive Ln-MOFs as optical markers for high-security anticounterfeiting technologies aimed to prevent illegal copies of sensitive identity documents, banknotes, diplomas, and certificates,^23−27^ which require an ever-increasing tunability (e.g., emission colors) and authentication complexity. However, regardless of the great potential of these materials, to date they have proved unsuitable for practical applications due to their limited chemical^28−35^ and/or optical^27,36^ stability under environmental conditions (e.g., humidity, temperature, etc.).

**Figure 1:**
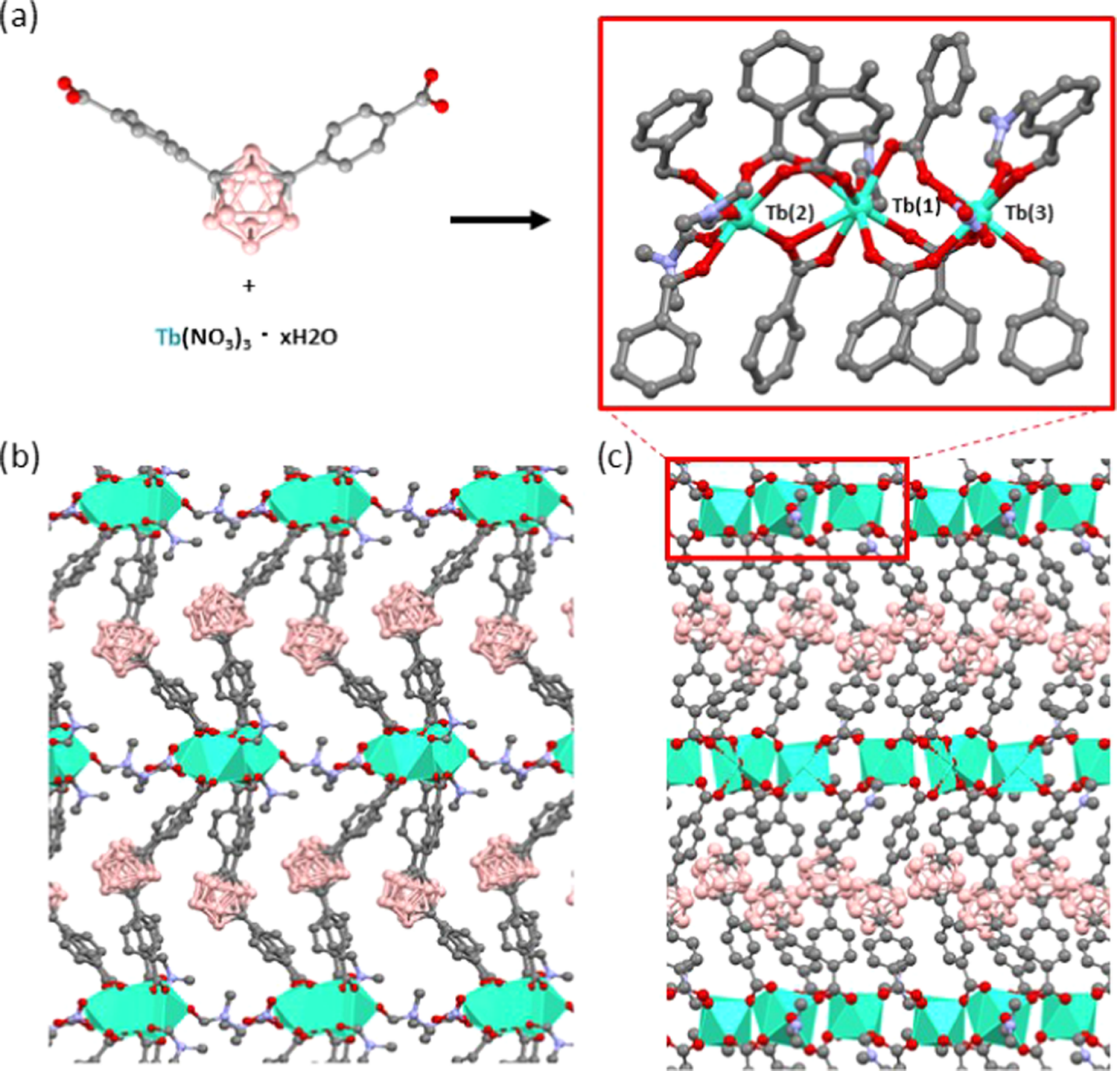
Crystal structure of ***m*****CB-Tb**. (a) View of the coordination of *m*CBL1 to the three independent Tb atoms that are repeated along the structure to provide one-dimensional (1D) inorganic rod-shaped chains and (b, c) two perpendicular views of the extended 3D framework along the *b* and *a* axes, respectively. Green polyhedra represent the Tb coordination spheres and H atoms are omitted for clarity. Color code: B, pink; C, gray; O, red; N, dark blue; and Tb, green.

Herein, we hypothesized that such limitations can be overcome with the introduction of carborane clusters such as icosahedral carboranes 1,*n*-C_2_B_10_H_12_ (*n* = 2, 7 or 12), a class of commercially available and exceptionally stable three-dimensional (3D) aromatic boron-rich clusters that possess material-favorable properties such as thermal/chemical stability and high hydrophobicity.^37−43^ Carborane-based MOFs were first synthesized at Northwestern University, and they showed an increase in their thermal stabilities among other interesting properties.^44−51^ The spherical nature of the carboranes, with slightly polarized hydrogen atoms and the presence of the hydride-like hydrogens at the B–H vertexes, make the carboranes very hydrophobic. Thus, we have recently explored and demonstrated the possibility of increasing the hydrolytic stability of CPs or MOFs by incorporating hydrophobic carborane-based linkers^52−57^ into these porous materials.^58−63^ Our strategy has provided the most water-stable Cu-paddle wheel MOF in the literature, which is related to the high hydrophobicity of the *m*-carborane ligand *m*CBL1: 1,7-di(4-carboxyphenyl)-1,7-dicarba-*closo*-dodecaborane (Figure 1).^61^ Beyond stability, the delocalized electron density is not uniform through the cage, giving rise to extraordinary differences in the electronic effects of the cluster.^64^ This unusual electronic structure is often highlighted by considering carboranes as inorganic three-dimensional “aromatic” analogues of arenes.^65^ In this regard, for the last 25 years, a remarkable influence of icosahedral carboranes on the photophysical properties of organic fluorophores^66−78^ or in their transition metal compounds has been reported.^57,79−81^ However, as far as we know, there are no reports on luminescence properties of carborane-based MOFs^82^ and therefore the antenna effect has not yet been reported for a carborane linker.

As a proof-of-concept, in this work, we report the preparation and full characterization of a series of isostructural water-stable *m*-carborane Ln-MOFs, {[(Ln)_3_(*m*CBL1)_4_(NO_3_)(DMF)*n*]·Solv} (***m*CB-Ln**, where **Ln** = **Eu**, **Tb**, or **Eu***_**x**_***Tb**_**1–*x***_; Figure 1). In addition to their high thermal and water stabilities, the preparation of mixed ***m*CB-Eu***_**x**_***Tb**_**1–*x***_-doped MOFs allowed for fine control and high tunability of both steady-state and time-dependent emission color (from green to red) and lifetime luminescence (from about 0.5 to 10 000 μs). An outstanding increase of 237% of luminescence quantum yield from the single-ion *m*CB-Eu MOF (20.5%) to the mixed *m*CB-Eu_0.1_Tb_0.9_ MOF (69.2%) is achieved, owing to a highly effective ET process from Tb^3+^ to Eu^3+^. Furthermore, the time-dependent luminescence of mixed MOFs and the typical discrete visible emission bands of Eu and Tb ions allowed for time-dependent bar-coding, whose code evolution in the ms scale can easily be tuned by controlling the Eu/Tb ratio. These advanced optical properties, combined with the demonstrated printability through spray-coating, make these materials very promising as invisible security inks for future anticounterfeiting technologies.

## Results and Discussion

### Syntheses, Characterization, and Optical Stability of Single-Ion Carborane-Based ***m*****CB-Ln**

Colorless crystals of [(Eu)_3_(*m*CBL1)_4_(NO_3_)(DMF)*_x_*]*_n_*·solv (***m*****CB-Eu**) and [(Tb)_3_(*m*CBL1)_4_(NO_3_)(DMF)*_x_*]*_n_*·solv (***m*****CB-Tb**) were obtained in high yields by solvothermal reactions in a mixture of *N*,*N*-dimethylformamide (DMF)/methanol/H_2_O at 95 °C for 48 h (see the Experimental Section for details and Figure S1, Supporting Information). Single-crystal X-ray diffraction revealed that ***m*****CB-Tb** crystallizes in the monoclinic *Pn* space group, and the analysis of the structure revealed the formation of a 3D framework based on the novel [(Tb)_3_(COO)_8_(NO_3_)(O_DMF_)_4_] secondary building unit (SBU) (Figure 1 and Table S1, Supporting Information).

The new SBU is composed of three nonequivalent crystallographic terbium atoms, which are connected and capped by bridging, chelate bridging or chelate *m*CBL1, chelate NO_3_^–^, and DMF molecules. Whereas, Tb(1) and Tb(3) atoms (Figure 1) are eight-coordinated and Tb(2) is seven-coordinated. As shown in Figure 1, six *m*CBL1 ligands are coordinated to Tb(1) and those adopt two different coordination modes (bridging and chelate bridging). The coordination of Tb(1) is completed by a DMF molecule. Tb(2) (Figure 1) shows, however, two coordinated DMF molecules and five mCBL1 ligands, all with bridging coordination. Tb(3) shows a DMF molecule, a chelate NO_3_^–^, and five *m*CBL1 ligands, the latter adopting bridging coordination with the neighboring Ln atoms. Such coordination provides 1D-chains of Tb atoms, which are connected by the *m*CBL1 ligands and thus provide the observed 3D structure (Figure 1). The varied coordination around the three crystallographic-independent Ln atoms results in three different Tb–Tb metal distances (Tb(1)–Tb(2) 5.5830(8), Tb(2)–Tb(3) 5.2550(7), and Tb(1)–Tb(3) 4.6398(7) Å). The Tb–O bond distances are in the range of 2.272(10)–2.906(10) Å, all of which are comparable to related compounds.^83−86^

Fourier transform infrared (FTIR) spectroscopy (Figure S2, Supporting Information) and powder X-ray diffraction (PXRD; Figure S3, Supporting Information) analysis for ***m*****CB-Eu** and ***m*****CB-Tb** compounds revealed that both are isostructural and their experimental patterns match very well with those simulated from the X-ray structure of ***m*****CB-Tb**, therefore, suggesting that the as-synthesized materials are pure phases. Thermogravimetric (TGA; Figure S4, Supporting Information) and elemental analyses confirmed the chemical composition of ***m*****CB-Eu** and ***m*****CB-Tb**. TGA curves for these two materials revealed good thermal stabilities as the frameworks are stable up to 400 °C.

As expected, both ***m*****CB-Eu** and ***m*****CB-Tb** showed very high stability in neutral water and aqueous solutions of a broad range of pH values (3–11) for at least 5 days. PXRD traces of both, before and after incubation in water in a closed vial perfectly match the simulated pattern derived from the single-crystal structure of ***m*****CB-Tb** (Figure S5, Supporting Information). In addition, optical images of the crystalline samples after their immersion in water under the above-mentioned conditions showed no significant morphology change in the needle-like crystals nor evidence of surface cracking (Figure S5, Supporting Information). Such high stability is ascribed to the presence of the carborane ligand.

**Figure 2:**
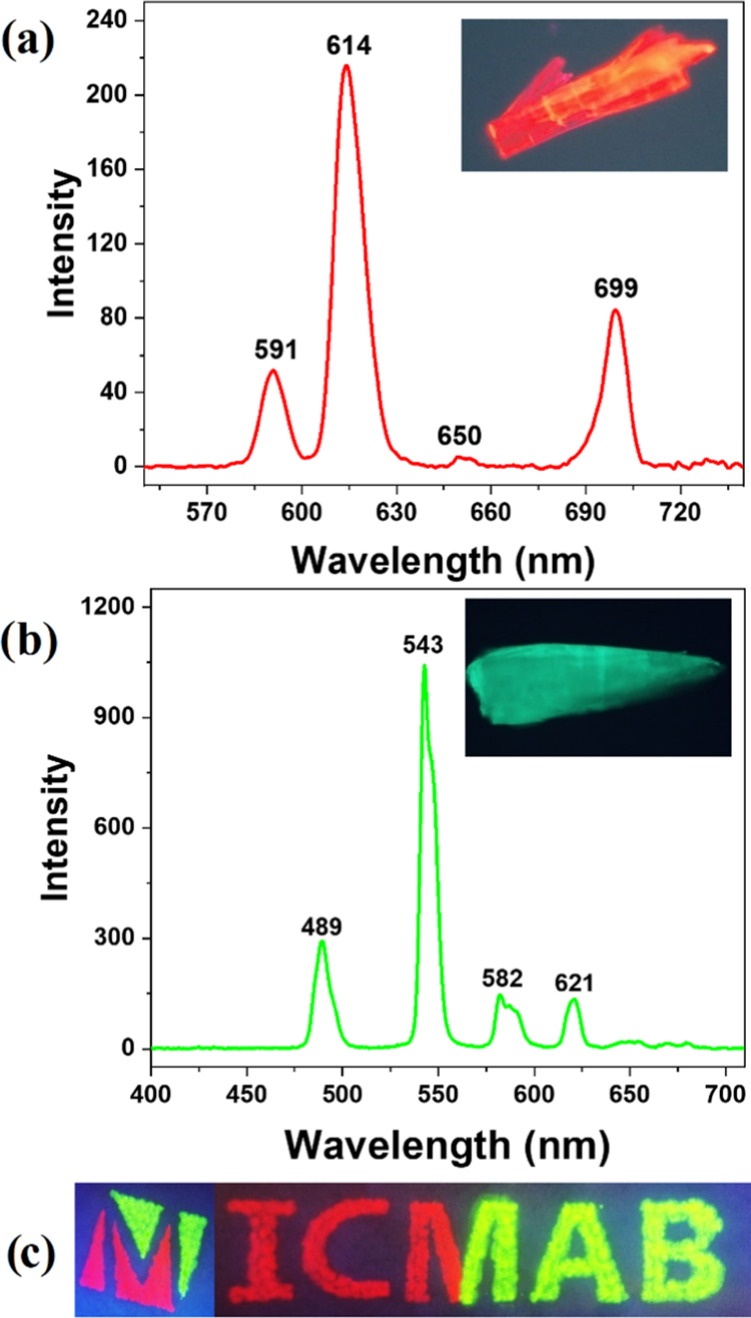
Solid-state emission spectra of ***m*****CB-Eu** (a) and ***m*****CB-Tb** (b) under continuous-wave irradiation (λ_ex_ = 280 nm) at room temperature. Insets: optical microscopy images of the corresponding crystals (λ_exc_ = 280 nm). (c) Photograph of the hand-painted logo of the Institut de Ciència de Materials de Barcelona (ICMAB) with ***m*****CB-Eu** and ***m*****CB-Tb** crystals (λ_ex_ = 254 nm).

The optical properties of the carborane-based *m*CBL1 ligand and the corresponding Eu^3+^ and Tb^3+^ compounds ***m*****CB-Ln** were investigated by collecting the ultraviolet–visible (UV–vis) absorption and emission spectra of the compounds in the solid state. The free ligand *m*CBL1 exhibits a broad absorption band around λ_max_ ∼ 289 nm attributed to π → π* transitions (Figure S6, Supporting Information). The luminescence spectrum for *m*CBL1 shows a strong emission at λ_em_ = 312 nm (λ_ex_ = 280 nm) and an overall quantum yield (Φ) of 0.3% (Figure S7, Supporting Information). The absorption spectra of ***m*****CB-Ln** display slight broadening of the UV bands. Upon continuous-wave irradiation at λ_ex_ = 280 nm in an air atmosphere and at room temperature, both ***m*****CB-Eu** and ***m*****CB-Tb** solid crystals showed intense luminescence in the visible region and sharp and well-resolved emission bands (Figure 2). The crystals’ emissions were also observable by the naked eye, as shown in the insets of Figure 2a,b. The luminescence spectrum of ***m*****CB-Eu** presented the typical emission feature of Eu-based materials, with peaks at 591, 614, 650, and 699 nm, which correspond to characteristic transitions of the Eu^3+^ ion: ^5^D_0_ → ^7^F*_J_* (*J* = 1, 2, 3, and 4),^87^ respectively, with the strongest being the ^5^D_0_ → ^7^F_2_ transition at 614 nm (Figure 2). Overall, the ***m*****CB-Eu** crystal yielded a strong orange luminescence quantum yield (Φ = 20.5%), with a 1931 CIE color coordinate (0.62, 0.38). ***m*****CB-Tb** showed the typical luminescence of the Tb^3+^ ion, with emission peaks at 489, 543, 582, and 621 nm, which are assigned to the ^5^D_4_ → ^7^F*_J_* (*J* = 6, 5, 4, and 3)^87^ transitions of Tb^3+^ ions. The strongest emission peak at 543 nm is associated with the ^5^D_4_ → ^7^F_5_ transition (Figure 2). ***m*****CB-Tb** presented a quite efficient green emission (Φ = 49.8%) with the CIE color coordinate (0.32, 0.58). These results clearly indicate that the carborane-based *m*CBL1 ligand is an excellent light-absorbing antenna chromophore for sensitizing both ions (vide infra), and the resulting MOFs presented quite high solid-state luminescence, which is comparable to other Ln-MOFs (Φ_Eu-MOFs_ = 25–95; Φ_Tb-MOFs_ = 7–75).^88−90^ More importantly, the optical properties of ***m*****CB-Eu** and ***m*****CB-Tb** crystals did not suffer significant changes when these materials were suspended in water for 5 days or heated up to 180 °C for 24 h (Figure S8, Supporting Information), proving the high stability provided by the carborane ligand to the MOF optical properties. In fact, water suspensions of the ***m*****CB-Eu** and ***m*****CB-Tb** crystals could be successfully used to prepare two-colored patterned luminescence drawings (of ICMAB logo) through their deposition onto cellulose papers (Figure 2c and the Experimental Section), which did not affect the emission properties. Scanning electron microscopy (SEM) images corroborate the entrapment of microsize crystals between the fibers of the cellulose papers (Figure S9, Supporting Information), and steady-state luminescence spectra demonstrate that the crystals preserve their optical properties (Figure S10).

**Figure 3:**
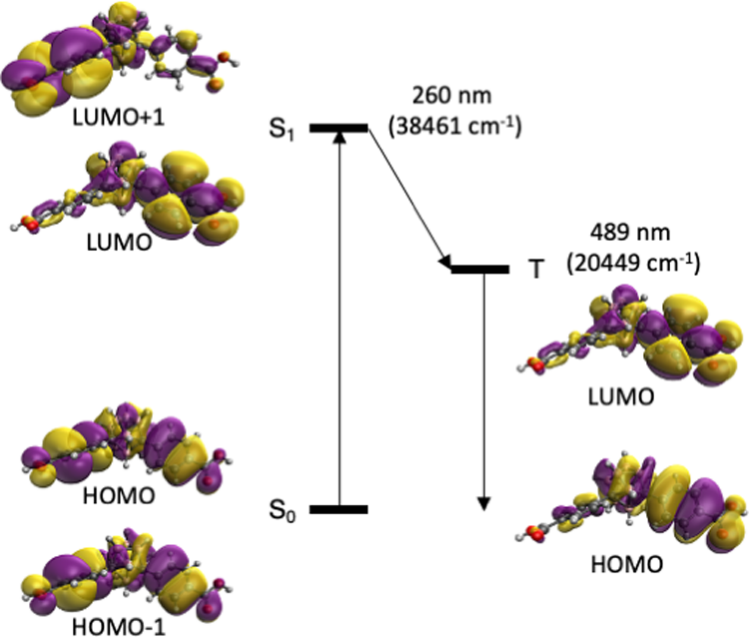
Energy diagram of the singlet and triplet states calculated using TDDFT with the B3LYP functional. The orbitals involved in such processes of the *m*CBL1 ligand are shown.

To analyze the mechanism of the luminescence process, the photochemical properties of the mCBL1 have been explored using time-dependent density functional theory (TDDFT) methods (see the Computational Details section). It is known that the antenna effect of the ligand for sensitization of the luminescence of lanthanide compounds is due to the transfer from a triplet state of the ligand to the first excited state of the lanthanide cataion .^14,16^ Among others, the efficiency of the ligand as a sensitizer is related to the energy of its triplet state. The energy of the ^5^D_4_ and ^5^D_0_ first excited states for Tb^3+^ and Eu^3+^ cations for the studied system are 541 nm (18 464 cm^–1^) and 614 nm (15 286 cm^–1^). To have an efficient energy transfer from the sensitizer ligand to the lanthanide, previous studies^14^ have estimated that the energy of the triplet of the ligand should be at least 1850 cm^–1^ above the lowest emitting excited states of the lanthanide. The first triplet state structure of the ligand has been optimized at the TDDFT level and resulted in a value of 20 449 cm^–1^ for *m*CBL1, which perfectly fits with the requirement for an efficient energy transfer to both Tb^3+^ and Eu^3+^. The involved energies in the first singlet excitation and the triplet energy of *m*CBL1 are represented in Figure 3 together with the involved orbitals. The first allowed excitation energies (calculated TDDFT values of 260 nm for the *m*CBL1 ligand) are in agreement with those determined in the *m*CBL1 ligand in a solid state around 251–289 nm (Figure S6, Supporting Information). The analysis of the orbitals confirms that such transitions are mainly π–π transitions with a large contribution from the phenyl rings. Both the calculated energies of the S_1_ and T_1_ states for *m*CBL1 are significantly larger than those for some commonly used carbon-based chromophores.^14^ Thus, to understand the possible role of the carborane moiety in such unusually high energies of the first singlet excitation and triplet for our ligand, we have also explored the photophysical properties of the related ligand by substituting the carborane moiety with a phenyl ring ([1,1′:3′,1″-terphenyl]-4,4″-dicarboxylic acid, *m*TDCA) by TDDFT (Figures S11 and S12, Supporting Information). Consistent with the previous reports,^14^ both the calculated energies of the S_1_ and T_1_ states for *m*TDCA are significantly smaller than that for *m*CBL1. The comparison between the two ligands (Figures S11 and S12, Supporting Information) shows that the main difference is a symmetry breaking of the empty orbitals, probably due to the smaller symmetry of the *m*CBL1 ligand for the central carborane. Whereas the *m*CBL1 ligand structure remains almost unchanged in the S_1_ and T_1_ states, that for the *m*TDCA ligand shows that the noncoplanar ground-state structure results in a two-ring coplanar for the first triplet state (Figure S12, Supporting Information). This difference between the two ligands is reflected in the emission energies of the triplet (*m*CBL1, 20449 cm^–1^; *m*TDCA, 16 474 cm^–1^). The orbitals involved in the emission are basically the lowest unoccupied molecular orbital (LUMO) and the highest occupied molecular orbital (HOMO) with a larger degree the localization in one part of the molecule in comparison with the singlet due to the decrease of symmetry (Figure 3). The unusually high energy for the triplet state for *m*CBL1, therefore, favored an effective energy transfer through nonradiated excited states of the metal until it reached the emissive levels and the metal-centered emission took place.^14^ Such energy transfer would be much less efficient in the case of the *m*TDCA ligand, which has no carborane, as the energy for its triplet state is of the order of that for Eu^3+^ and lower than that for Tb^3+^ (Figure S13, Supporting Information).

### Synthesis, Characterization, and Optical Properties of Mixed-Ion Carborane-Based ***m*****CB-Eu***_**y**_***Tb**_**1–*y***_

Currently, doping diverse Ln^3+^ ions into the same MOF has become an emerging method to accomplish stoichiometry-dependent color tunability.^16,90,91^ Due to the similar coordination environments, various Ln^3+^ ions can be introduced into the same MOF structure simultaneously. Energy transfer (ET) from one lanthanide to another lanthanide ion has also been observed to enhance the luminescence intensity in mixed-metal Ln-MOFs.^88,91−96^ For example, it has been reported that such ET between Tb and Eu ions induced up to 70% emission enhancement for the Tb-sensitized Eu emission in Ln-MOFs.^88^ Thus, after once demonstrating the feasibility of using the hydrophobic carborane ligand to obtain water-stable MOFs with a high luminescence quantum yield, we aimed to investigate the possibility of obtaining other mixed Ln-MOFs (***m*****CB-Eu***_**y**_***Tb**_**1–*y***_) with variable amounts of each lanthanide, which are also expected to provide different luminescence colors. [(Eu*_y_*Tb_1–*y*_)_3_(*m*CBL1)_4_(NO_3_)(DMF)*_x_*]*_n_*·solv (***m*****CB-Eu***_**y**_***Tb**_**1-*y***_) were obtained as needle-like crystals (Figure S1, Supporting Information) and in good yields (>64%) by following the solvothermal procedure employed for the single-ion MOFs (see the Experimental Section for details). PXRD spectra for all ***m*****CB-Eu***_**y**_***Tb**_**1–*y***_ compounds match very well with the individual ***m*****CB-Eu** and ***m*****CB-Tb** counterparts and therefore also proved to be also isostructural (Figure S3). The Eu/Tb molar ratios in the mixed MOFs were determined by inductively coupled plasma (ICP) measurements, revealing that the ratios match reasonably well with the original molar ratios of Eu^3+^/Tb^3+^ during the syntheses (Table S2).

**Figure 4:**
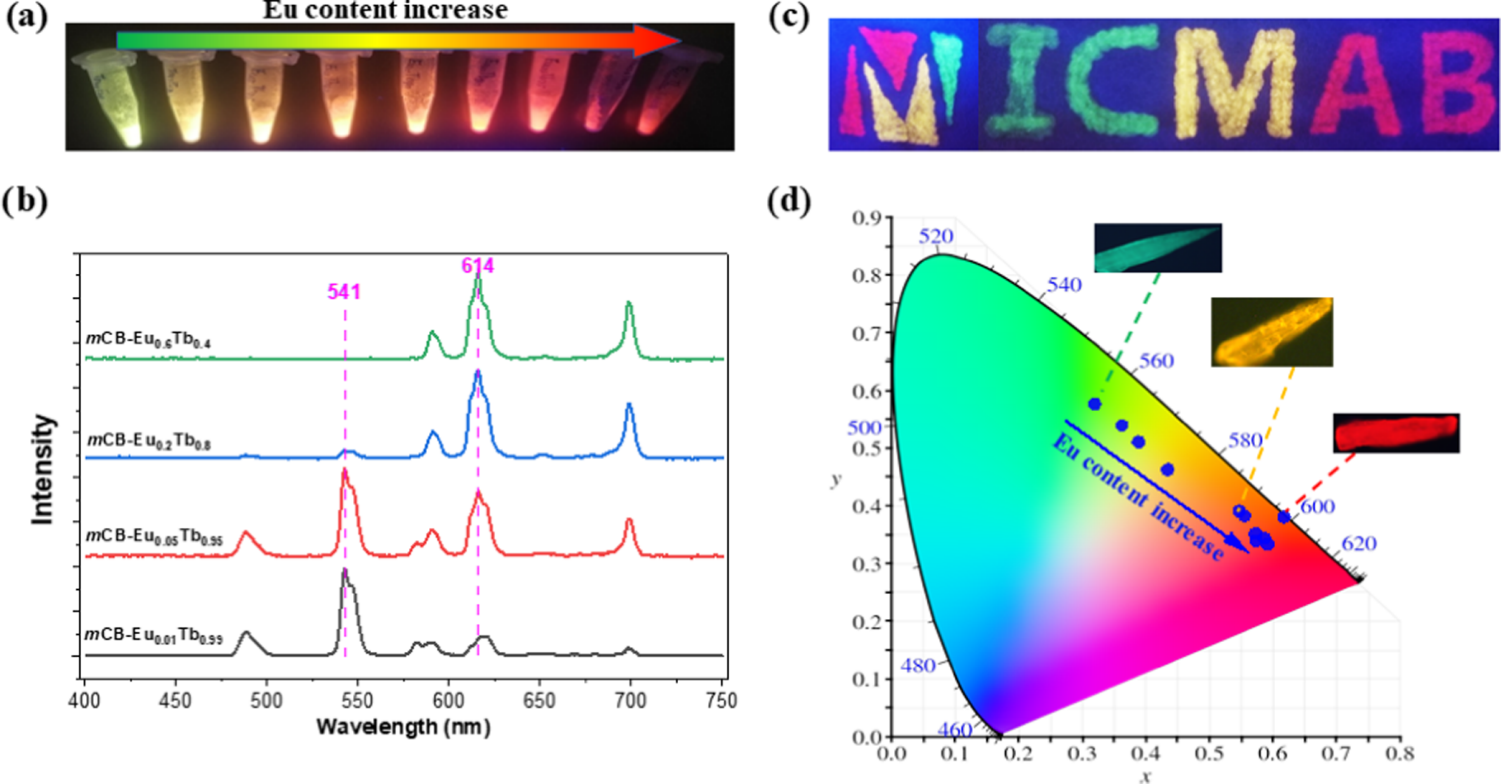
(a) Photographs of the powders of the mixed ***m*****CB-Eu***_**y**_***Tb**_**1–*y***_ (λ_ex_ = 254 nm); (b) selection of steady-state emission spectra of the powders of mixed ***m*****CB-Eu***_**y**_***Tb**_**1–*y***_ with various Eu/Tb molar ratios (λ_ex_ = 280 nm) (see Figure S14 for the spectra of all ***m*****CB-Eu***_**y**_***Tb**_**1–*y***_ series); (c) photograph of the hand-painted logo of the Institut de Ciència de Materials de Barcelona (ICMAB) with ***m*****CB-Tb** (green), ***m*****CB-Eu**_**0.1**_**Tb**_**0.9**_ (yellow), and ***m*****CB-Eu** (red) crystals; and (d) color coordinates drawn onto the 1931 CIE chromaticity diagram for the mixed ***m*****CB-Eu***_**y**_***Tb**_**1–*y***_. Inset: luminescence microscopy images of the ***m*****CB-Tb** (green), ***m*****CB-Eu**_**0.1**_**Tb**_**0.9**_ (yellow), and ***m*****CB-Eu** (red) crystals.

Steady-state irradiation (λ_ex_ = 280 nm) of the obtained solid ***m*****CB-Eu***_**y**_***Tb**_**1–*y***_ crystal powders yielded strong emission in the visible spectral region in all cases, with the emitted color finely and fully tunable between the two extreme colors (green and red) of the single-element Ln-MOFs (Figure 4a).

A detailed analysis of the luminescence data for ***m*****CB-Eu***_**y**_***Tb**_**1–*y***_ crystals (Table 1, Figures 4b and S14, Supporting Information) discloses some interesting results. On the one hand, the increase in the molar fraction of Eu from 0 to 0.08 in the mixed Ln-MOF preparation caused a quite significant and gradual shift of the emission color, from green (***m*****CB-Tb**) to orange (Figure 4b), as shown also by the corresponding CIE color coordinates and representative luminescence microscopy images (inset Figure 4d). However, a further increase of the Eu percentage (up to 100%) yielded less variation in the emission ratios of the two elements in ***m*****CB-Eu***_**y**_***Tb**_**1–*y***_ and thus a less significant color change toward the red region of ***m*****CB-Eu**. This was ascribed to the negligible Tb emission contribution in the ***m*****CB-Eu***_**y**_***Tb**_**1–*y***_ crystals above a threshold Eu amount (10%), as a consequence of an efficient Tb^3+^ energy transfer to Eu^3+^ (Table 1).

**Table 1:** CIE Color Coordinates, Luminescence Lifetimes, Energy Transfer Efficiencies, Absolute Quantum Yield, and Emission Ratio for ***m*****CB-Eu**, ***m*****CB-Tb**, and ***m*****CB-Eu***_**y**_***Tb**_**1–*y***_ (λ_ex_ = 280 nm)

		τ (μs)^a^				
Ln	CIE color coordinates	(^5^D_4_ of Tb^3+^)	(^5^D_0_ of Eu^3+^)	η_Tb→Eu_^b^ (%)	Φ (%)	emission ratio of Eu/Tb
Eu	(0.62, 0.38)		739.0		20.5 ± 1.3	1.000/0.000
Eu_0.6_Tb_0.4_	(0.59, 0.33)	23.2	749.7	97.3	41.2 ± 2.1	0.997/0.003
Eu_0.5_Tb_0.5_	(0.58, 0.34)	60.2	859.6	92.9	42.5 ± 1.4	0.974/0.026
Eu_0.25_Tb_0.75_	(0.59, 0.34)	117.9	934.1	86.1	47.8 ± 2.0	0.971/0.029
Eu_0.2_Tb_0.80_	(0.57, 0.35)	219.3	1023.9	74.2	58.1 ± 2.8	0.949/0.051
Eu_0.1_Tb_0.90_	(0.58, 0.38)	331.6	1079.6	61.0	69.2 ± 2.6	0.849/0.151
Eu_0.08_Tb_0.92_	(0.55, 0.39)	465.1	1084.1	45.3	63.6 ± 2.3	0.825/0.175
Eu_0.05_Tb_0.95_	(0.44, 0.46)	575.0	1153.8	32.3	56.4 ± 2.7	0.516/0.484
Eu_0.03_Tb_0.97_	(0.39, 0.51)	676.4	1367.3	20.4	55.7 ± 1.7	0.248/0.752
Eu_0.01_Tb_0.99_	(0.36, 0.54)	818.3	1714.9	3.7	52.6 ± 2.5	0.230/0.770
Tb	(0.32, 0.58)	849.7			49.8 ± 1.8	0.000/1.000

a Decay curves for mixed Ln-MOFs were fitted by a biexponential function (*I* = *A*_1_ exp(−*t*/τ_1_) + *A*_2_ exp(−*t*/τ_2_)), and the average lifetime was calculated from the equation of τ = (*A*_1_τ_1_^2^ + *A*_2_τ_2_^2^)/(*A*_1_τ_1_ + *A*_2_τ_2_).^22,97^

b Energy transfer efficiency was determined by the function of η_Tb→Eu_ = 1 – τ/τ^0^.^96^

**Figure 5:**
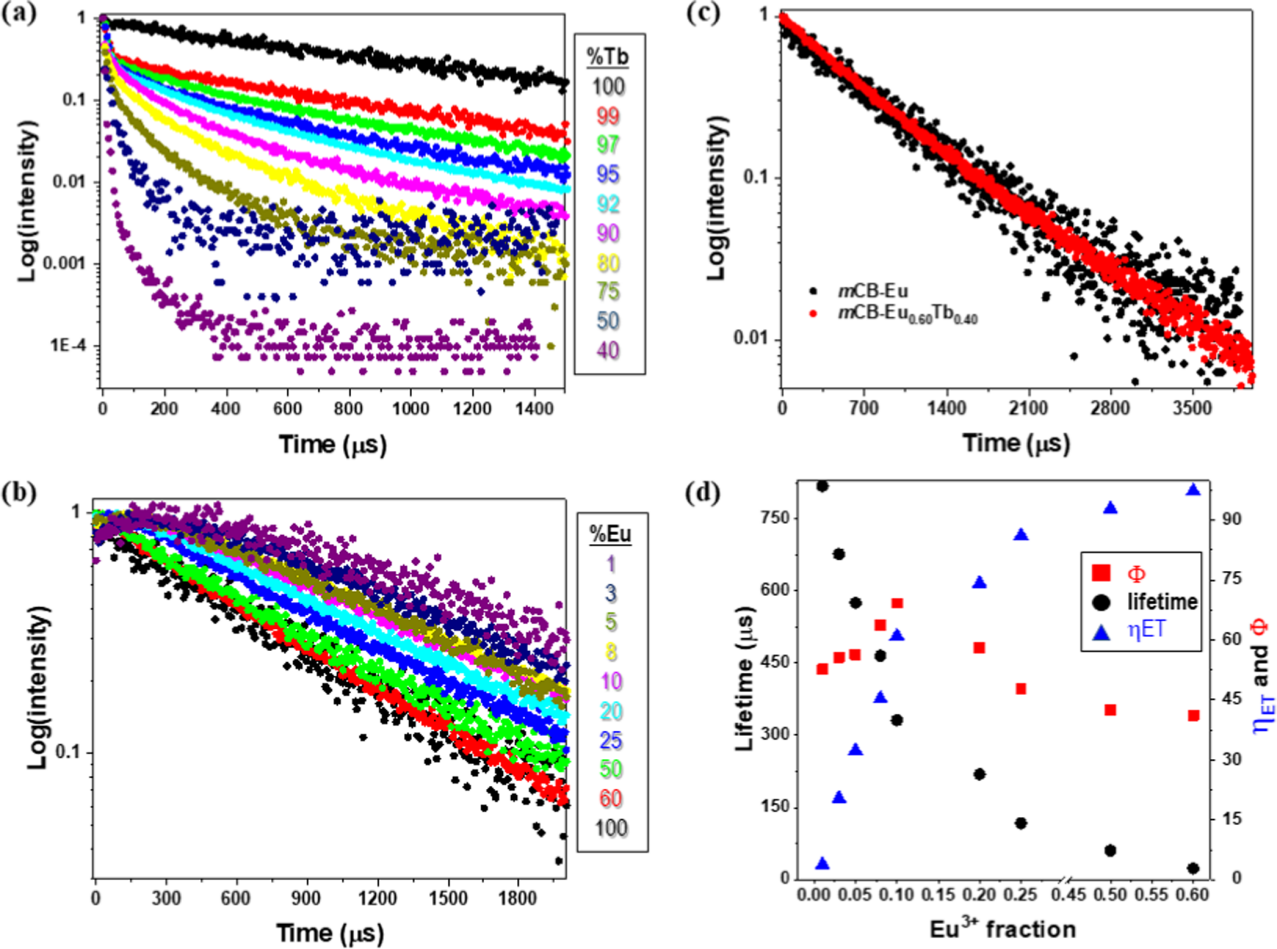
(a) Luminescence decays of Tb (λ_em_ = 541 nm) in the different MOFs (λ_exc_ = 280 nm); (b) luminescence decays of Eu (λ_em_ = 614 nm) in the different MOFs (λ_exc_ = 280 nm); (c) comparison of the luminescence decay of mCB-Eu_0.6_Tb_0.4_ with *m*CB-Eu; and (d) average lifetimes, ET quantum yield, and luminescence quantum yield trends against the Eu^3+^ fraction.

These results indicate that the tunable emission in the ***m*****CB-Eu***_**y**_***Tb**_**1–*y***_ crystals is not only related to the additive relative luminescence of the Eu^3+^ and Tb^3+^ component elements but is also the result of efficient energy transfer processes from Tb^3+^ to Eu^3+^,^88,91−96,98^ which results in an enhancement of the of Eu^3+^ luminescence instead of additive emissions from each ion. These mixed-ion MOF crystals were successfully employed to prepare multicolored patterned luminescence drawings via hand-painting onto cellulose papers, which are colorless (i.e., invisible crystals) under ambient light, while preserving the emission properties under UV radiation, making them highly suitable for anticounterfeiting technologies (Figure 4c). Further evidence of the Tb-Eu ET process is derived from the study of the luminescence decays of Tb^3+^ and Eu^3+^ ions in all of the above compounds, registered at λ_em_ = 541 (^5^D_4_ → ^7^F_5_ of Tb^3+^) and 614 nm (^5^D_0_ → ^7^F_2_ of Eu^3+^), respectively, upon pulsed light irradiation at λ_exc_ = 280 nm (Figures 5 and S15–26, Supporting Information).

The luminescence decay curves of ***m*****CB-Tb** and ***m*****CB-Eu** exhibited the typical monoexponential decay functions with calculated lifetimes of 849.7 and 739.0 μs, respectively, which are similar to those reported for other Eu- and Tb-based compounds (Table 1 and Figure S15, Supporting Information).^90^ The emission decay curves of Tb^3+^ in ***m*****CB-Eu***_**y**_***Tb**_**1–*y***_, changed to biexponential decay functions, with increasingly shorter average decay times (Table 1 and Figures 5a and S16, Supporting Information). In contrast, in the emission curve of Eu^3+^ of the mixed-metal MOFs, a signal increase at shorter times is followed by a luminescence decrease. The increasing signal is slow for ***m*****CB-Eu**_**0.01**_**Tb**_**0.99**_, but becomes shorter (i.e., faster) as the Eu^3+^ concentration increases, in good agreement with the lifetime decrease of the Tb^3+^ (Figures 5b and S17). The apparent increase of Eu^3+^ lifetimes at smaller concentrations is the result of the convolution of the Eu^3+^ formation and its luminescence decay. In ***m*****CB-Eu**_**0.6**_**Tb**_**0.4**_, the signal rise is so fast that the measured decay matches with the monoexponential decay function and lifetime of the pure Eu^3+^ MOF (Figure 5c). These results corroborate the ET process between the Tb^3+^ and Eu^3+^, which becomes more efficient as the concentration of Eu^3+^ increases, becoming nearly quantitative (97.3%) in ***m*****CB-Eu**_**0.6**_**Tb**_**0.4**_ (Figure 5c).

**Figure 6:**
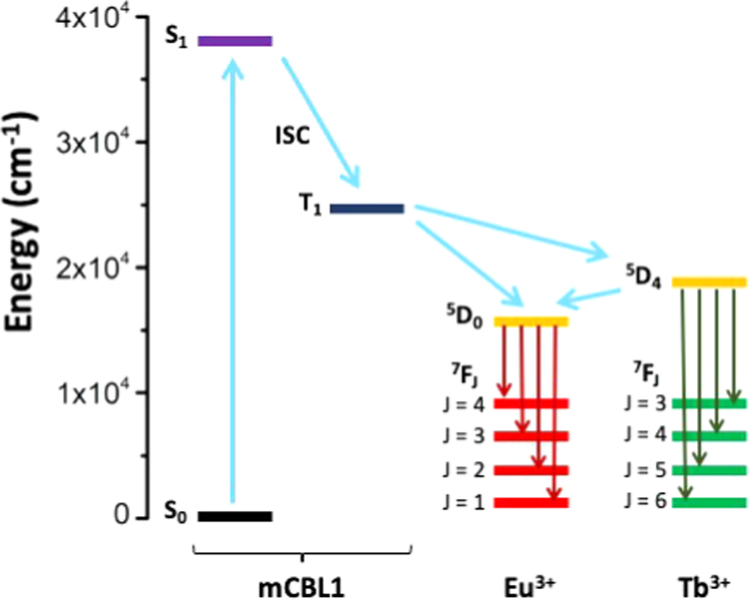
Schematic diagram of the energy absorption to the singlet state (S_0_) of the *m*CBL1 ligand, transfer to the triplet state (T_1_), energy transfer, and emission processes of ***m*****CB-Eu***_**y**_***Tb**_**1–*y***_.

Remarkably, the absolute quantum yields for ***m*****CB-Eu***_**y**_***Tb**_**1–*y***_ did not follow the same trend and varied greatly within these mixed-metal MOFs: it increased passing from the values of the single-ion crystals ***m*****CB-Tb** (49.8%) and ***m*****CB-Eu** (20.5%) up to a maximum of 69.2% in ***m*****CB-Eu**_**0.1**_**Tb**_**0.90**_ (Table 1 and Figure 5d), which represents an outstanding increase of 237% of the quantum yield of that for ***m*****CB-Eu** (20.5%) or an increase of 39% with respect to that for ***m*****CB-Tb** (49.8%). Such a huge enhancement of the overall quantum yield reveals that the ligand-to-Tb^3+^ and Tb^3+^-to-Eu^3+^ consecutive energy transfers are much more efficient than the direct ligand-to-Eu^3+^ energy transfer. However, when the Eu^3+^ amount increases significantly (20%), it starts competing with Tb^3+^ in the ET transfer from the ligand, lowering the overall quantum yield (Figure 6). The observed energy transfer process is well known to happen within these two metals, although such an increase in the quantum yield has not been reported.^88,91−96^

### Time-Dependent Optical Properties of ***m*****CB-Eu***_**y**_***Tb**_**1–*y***_ Crystals for Anticounterfeiting

A precise tailoring of a lifetime at an emission band can entail a virtually unlimited number of unique temporal codes.^99^ However, to date, a few reports have considered Ln-MOFs for lifetime-based encoding in the visible range for optical multiplexing,^27,98,100^ but tunable fluorescent lifetime has not been proposed for anticounterfeiting. The energy transfer process between Eu^3+^ and Tb^3+^ ions and the control of their decay rates in different ***m*****CB-Eu***_**y**_***Tb**_**1–*y***_ crystals allowed us to explore two optical features of interest for anticounterfeiting technologies for the first time: time-dependent emission color change and time-dependent bar-coding. These were demonstrated for ***m*****CB-Eu**_**0.01**_**Tb**_0.99_, ***m*****CB-Eu**_**0.1**_**Tb**_**0.90**_, and ***m*****CB-Eu**_**0.60**_**Tb**_**0.40**_ crystals where the luminescence lifetime variation of Eu^3+^ and Tb^3+^ is achieved by changing the metal stoichiometry in ***m*****CB-Eu***_**y**_***Tb**_**1–*y***_.

### Time-Dependent Emission Spectra (and Color)

**Figure 7:**
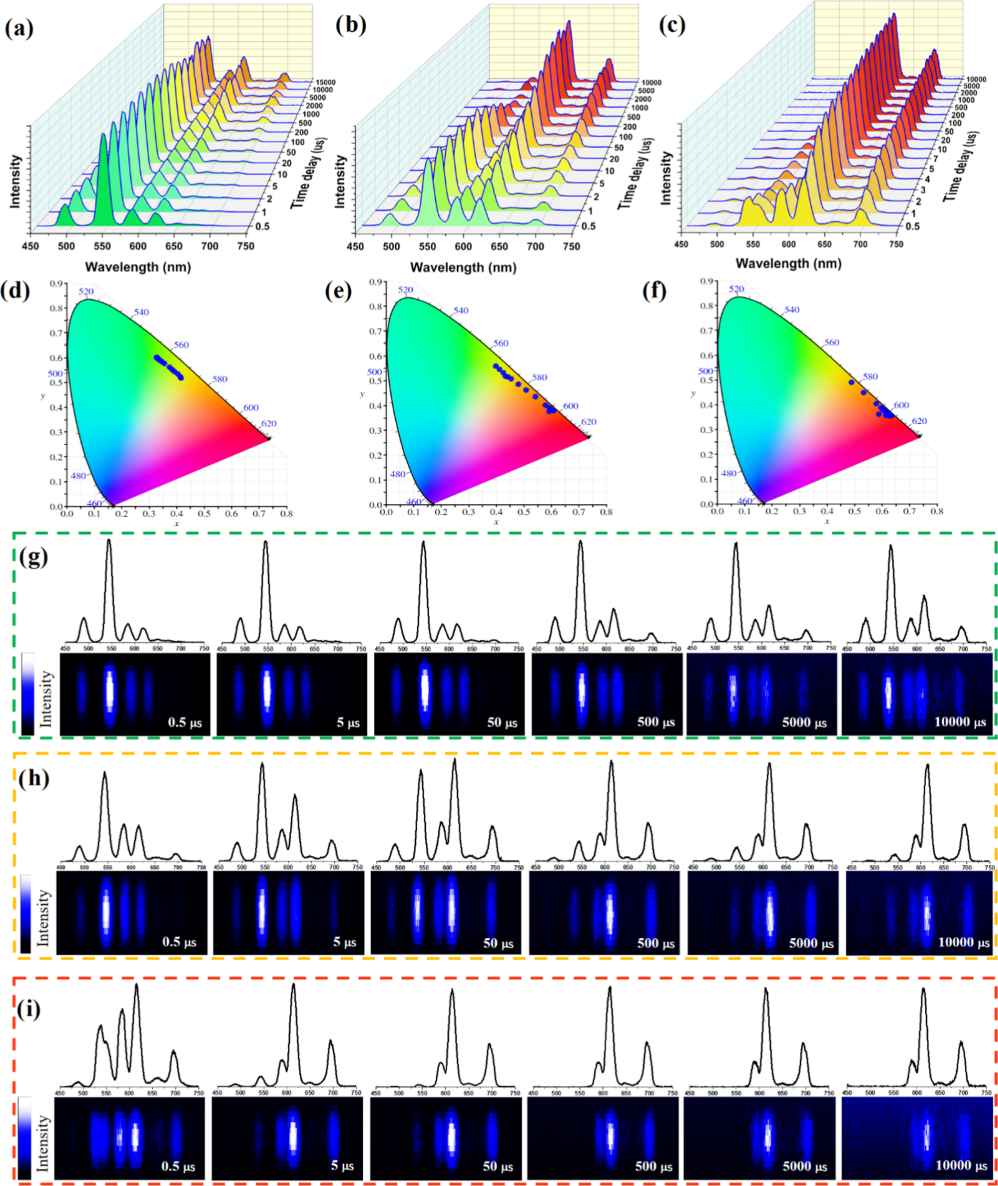
Time-dependent emission spectra of (a) ***m*****CB-Eu**_**0.01**_**Tb**_**0.99**_, (b) ***m*****CB-Eu**_**0.1**_**Tb**_**0.9**_, and (c) ***m*****CB-Eu**_**0.6**_**Tb**_**0.4**_ powders at various time delays and (d–f) corresponding CIE coordinates (λ_ex_ = 266 nm). Time-dependent bar codes of (g) ***m*CB-Eu_0.01_Tb_0.99_**, (h) ***m*CB-Eu_0.1_Tb_0.9_**, and (i) ***m*CB-Eu_0.6_Tb_0.4_** (λ_ex_ = 355 nm).

These were recorded at various delay times (0.5–10 000 μs) upon irradiation with a 266 nm pulsed neodymium-doped yttrium aluminum garnet (Nd-YAG) laser (Figure 7a–f). The spectra measured for the ***m*****CB-Eu**_**0.1**_**Tb**_**0.90**_ crystals gradually changed from green (0.40, 0.56) to red (0.61, 0.37) (Figure 7b), following a similar color variation trend registered under continuous-wave irradiation for MOFs made of different ion compositions. In this case, different colors were obtained from a single MOF at different delay times. The green color observed at shorter delays was due to the Tb^3+^ emission, which was still not quenched efficiently by Eu^3+^. The red color recorded at longer delays was ascribed to the Eu^3+^ emission after Tb^3+^ was fully quenched (complete ET). The intermediate colors were the result of the contribution of both the sensitized Eu^3+^ and the still not quenched Tb^3+^. When the same study was carried out for the ***m*****CB-Eu**_**0.01**_**Tb**_**0.99**_ powder, time-dependent emission spectra were still recorded, though they yielded greener coordinates at shorter delay times (0.33, 0.60) and a yellow color at longer delays (0.42, 0.52). The different range of color variation for this compound was ascribed to the slower Tb^3+^–Eu^3+^ energy transfer process that delays the loss of the green emission of Tb^3+^, which thus contributes significantly to the overall spectra until the end of the luminescence (Figure 7a). The opposite effect was observed for the ***m*****CB-Eu**_**0.60**_**Tb**_**0.40**_ crystals, which showed time-dependent emission spectra changing from yellow (0.49, 0.49) to red (0.59, 0.36), as shown in Figure 7c. In this case, by the time the first spectrum is recorded, the emission contribution of Tb^3+^ is already partially merged with the sensitized Eu^3+^ emission, providing the yellow coloring. Moreover, Tb^3+^ emission is, in this case, quickly quenched, yielding delayed emission spectra with only the red contribution of Eu^3+^. Therefore, these materials not only show time-dependent emission spectra and color coordinates but also enable the emission color range to be changed (and the starting and ending point) by simply modifying the stoichiometry of the ions. This time-dependent color change, observed in all mixed ***m*****CB-Eu***_**y**_***Tb**_**1–*y***_, introduces more complexity to the luminescence color tunability through the relative proportion of Eu^3+^/Tb^3+^.

### Time-Dependent Bar-Coding

For the second feature, we explored the possibility of using the discrete and narrow emission bands of lanthanide ions to obtain time-dependent bar-coding. For this, we performed pulsed measurements of the three compounds above, recording the projection of the emitted photons over time onto the detecting matrix of a charge-coupled device (CCD) camera. Taking advantage of the different decay profiles and rates of Tb^3+^ (emission decrease after the pulse) and Eu^3+^ (increase and then decrease), luminescent bar-coding changing the number and relative intensities of the lines (associated with the emitted bands of the two ions) was obtained (Figures 7g–i and S27–S29, Supporting Information). It is easy to understand the relevance of this time-dependent bar-coding to create dynamic security messages and labels changing the provided information in a microsecond–millisecond time scale. For ***m*****CB-Eu**_**0.1**_**Tb**_**0.90**_, the initial and final bar-code lines are related to the emission bands of nearly pure Tb^3+^ or Eu^3+^, respectively (Figures 7h and S28, Supporting Information). This means that the coded information changes all of the time along the recorded time frame. In the case of ***m*****CB-Eu**_**0.01**_**Tb**_0.99_ (slower ET), the emission of Eu^3+^ only starts appearing after 500 μs, which means the coded information only starts changing at later delays (Figures 7g and S27, Supporting Information). Finally, for the ***m*****CB-Eu**_**0.60**_**Tb**_**0.40**_ powder (fast ET), the Tb^3+^ lines only slightly appear for the first few microseconds, which means the coding information will not change further after a short delay time (Figures 7i and S29, Supporting Information). Similar results were obtained on irradiating with lower-energy excitation wavelengths (355 nm, Figures S30 and S32, Supporting Information).

**Figure 8:**
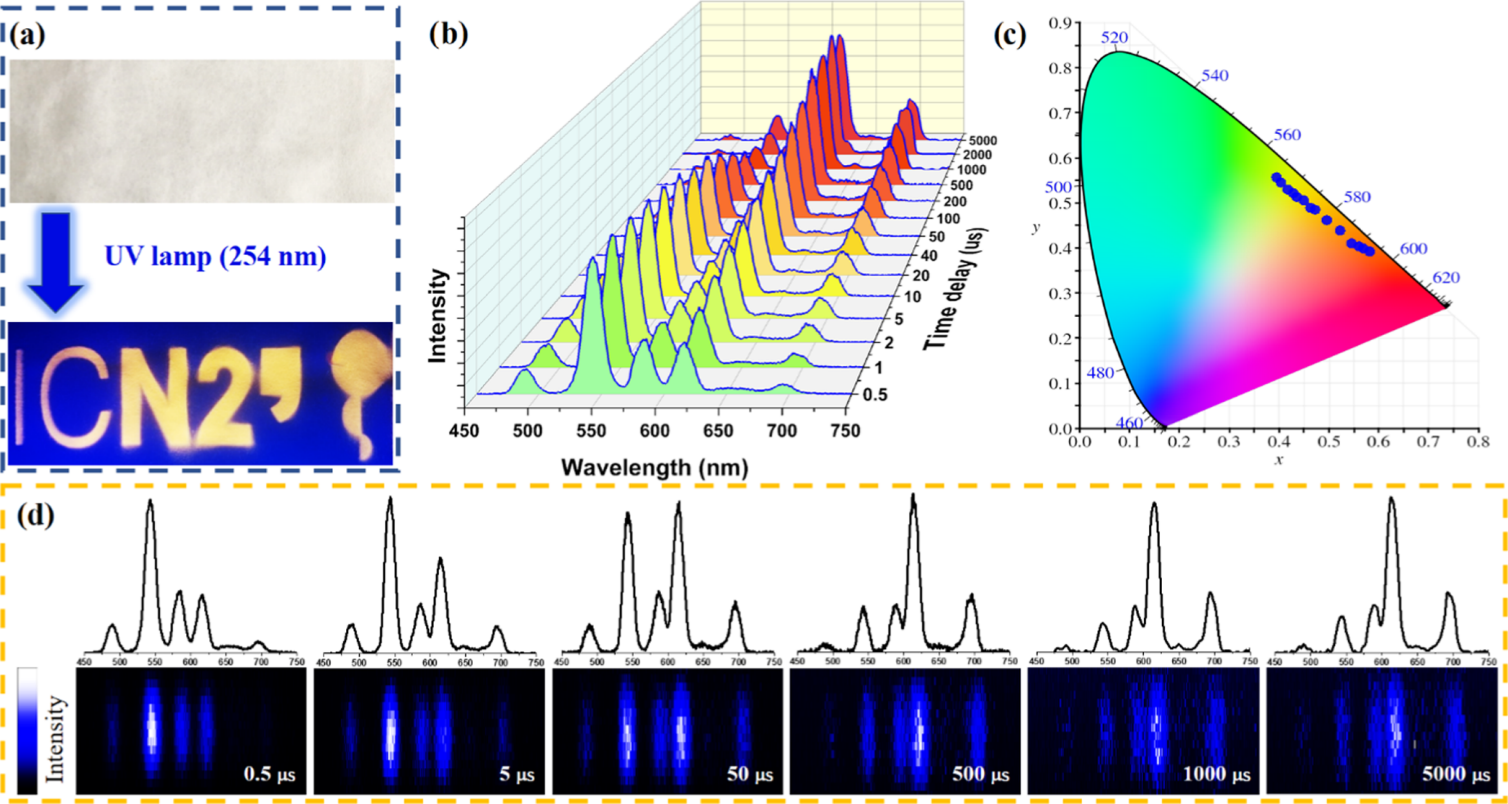
(a) Spray-coated ***m*****CB-Eu**_**0.01**_**Tb**_**0.99**_ using a prepatterned mask to illustrate the logo on Institut de Nanociencia i Nanotecnologia, (b) time-dependent emission spectra of the printed ***m*****CB-Eu**_**0.01**_**Tb**_**0.99**_, (c) corresponding color coordinates in the 1931 CIE diagram, and (d) time-dependent bar codes of the printed mCB-Eu_0.01_Tb_0.99_ (λ_ex_ = 355 nm).

These very promising results pushed us to use these encoding materials for printing onto cellulose papers to simultaneously obtain time-dependent luminescent colors and codes onto patterned spatial domains, which will bring new schemes for advanced anticounterfeiting technologies and security data storage. Printing was carried out through a custom-made spray-coating technique in which a prefabricated mask with a logo was layered onto the substrate under the nozzle (Figure S33, Supporting Information). The printed colorless cellulose paper showed no patterns under daylight while preserving the emission properties under UV radiation. Under continuous-wave UV irradiation, the printed pattern can be recognized (Figure 8a). The recorded steady-state emission spectra (Figure S34) yielded an orange color with coordinates at (0.57, 0.38) in the CIE 1931 color space diagram as the crystal powder (Figure 4b). However, measurements under pulsed irradiation (266 nm) revealed time-dependent luminescent spectra (Figure 8b), color (Figure 8c), and bar codes (Figures 8d and S31), confirming that the optical properties of the powder are preserved after printing in cellulose papers, the substrate most used for sensitive documents.

## Conclusions

Herein, we report carborane ligand-based Ln-MOFs (also known as lanthanide coordination polymers [Ln-CPs]) as a novel class of water- and temperature-stable materials that exhibit multimodal luminescence tunability. We have synthesized and fully characterized a new family of isostructural ***m*****CB-Eu***_**y**_***Tb**_**1–*y***_ (*y* = 0–1) luminescence MOFs built on a highly hydrophobic carborane linker (*m*CBL1). Ln-MOFs prepared from Eu^3+^ and Tb^3+^ at different ratios permitted easy modulation of the luminescence from the green to the red region of the 1931 CIE lab space diagram. This color tunability was ascribed to the controlled energy transfer (ET) efficiency between Tb^3+^ and Eu^3+^. The ET process was corroborated by both spectral measurements and lifetime decays, which showed nearly quantitative ET efficiency when the Eu^3+^ was increased to 60%. The different lifetimes of Tb^+^ and Eu^3+^ in each MOF also allowed time-dependent spectral changes in the ms time scale to be obtained. An outstanding increase of 237% of the quantum yield of *m*CB-Eu (20.5%) in the mixed ***m*****CB-Eu**_**0.1**_**Tb**_**0.9**_ (69.2%) is achieved, along with an increased and tunable lifetime luminescence (from about 10 to 10000 μs), all of these promoted by a highly effective ET process. Moreover, taking advantage of the narrow bands of Ln, we were able to obtain time-dependent bar codings, whose bars and the rate of change could be modulated by the MOF composition.

These results, together with the fact that these particles could be printed through spray-coating, make these materials highly attractive for dynamic color-changing security inks in anticounterfeiting technologies.

## Experimental Section

All chemicals were of reagent-grade quality. They were purchased from commercial sources and used as received. A 1,7-di(4-carboxyphenyl)-1,7-dicarba-*closo*-dodecaborane ligand (*m*CBH_2_L1) was synthesized according to the literature procedure.^101^

### Synthesis of {[(Ln)_3_(mCBL1)_4_(NO_3_)(DMF)_*n*_]·Solv} (**mCB-Ln**, Where **Ln = Eu**, **Tb**, and **Eu***_**x**_***Tb**_**1–*x***_)

The *m*CB-Ln materials were prepared by solvothermal synthesis. In a typical preparation, *m*CBH_2_L1 (0.03 mmol) and Ln(NO_3_)_3_ (0.02 mmol; Ln = Eu, Ln) were added to a mixture of DMF (0.5 mL)/methanol (1.5 mL)/H_2_O (0.3 mL) and sonicated until complete dissolution of all reagents. The above mixture was transferred to an 8 dram vial and heated at 95 °C in an oven for 48 h. Needle-like white crystals were collected and washed with DMF (yield based on the lanthanides: 71% for ***m*****CB-Tb** and 64% for ***m*****CB-Eu**). IR (ATR; selected bands; cm^–1^): 2601 (BH); 1658 (C=O from DMF); and 1590 (C=O from carboxylate). Elemental analysis (%) calculated for [Eu_3_(*m*CB-L)_4_(NO_3_)(DMF)_2_]·6H_2_O: C 36.23, H 4.14, N 2.07; found: C 36.36, H, 4.24, N 1.82. Elemental analysis (%) calculated for [Tb_3_(*m*CB-L)_4_(NO_3_)(DMF)_2_]·6H_2_O: C 36.23, H 4.14, N 2.07; found: C 36.16, H, 4.31, N 1.66.

The mixed ***m*****CB-Eu***_**x**_***Tb**_**1–*x***_ materials were prepared using the same method by adjusting the ratios of Eu(NO_3_)_3_/Tb(NO_3_)_3_ salts.

### Preparation of the Multimodal Anticounterfeiting Model

Anticounterfeiting tags were painted using fluorescent inks with an optimized concentration of Ln-MOFs (0.2 mg/mL). To prepare the aqueous security inks, crystals of Ln-MOFs were manually ground and then dispersed in water with the help of ultrasonication. A commercially available filter paper was used as a substrate in this study. A handwritten image was obtained using a stick contaminated with the security inks. To get a more regular printing pattern, a custom-made spray-coating technique was employed, in which a prefabricated mask with a logo was layered onto the substrate under the nozzle (Figure S31). It should be mentioned that the luminescence intensity of the inks and the subsequently printed patterns could be easily adjusted by varying the concentration of Ln-MOFs.

#### Instruments and Characterization

A crystal suitable for single-crystal X-ray diffraction (SCXRD) with dimensions 0.18 × 0.07 × 0.04 mm^3^ was selected and mounted on a MITIGEN holder with silicon oil on a ROD, Synergy Custom system, HyPix diffractometer. The crystal was kept at a steady *T* = 100(2) K during data collection. The structure was solved with the ShelXT 2014/5^102^ solution program using dual methods and using Olex2 1.5-α^103^ as the graphical interface. The model was refined with ShelXL 2016/6^104^ using full-matrix least-squares minimization on *F*^2^. The structure is refined in the monoclinic space-group *Pn* with a β angle of 90.094(1)° and a twin law replicating orthorhombic symmetry (100, 01̅0, 001̅), BASF = 0.43. The DMF molecules were refined as rigid groups with various thermal parameter restraints. Attenuated total reflection Fourier transform infrared (ATR-FTIR) spectra were recorded using a PerkinElmer Spectrum One spectrometer equipped with a Universal ATR sampling accessory. Spectra were collected with a 2 cm^–1^ spectral resolution in the 4000–650 cm^–1^ range. Elemental analyses were obtained using a Thermo (Carlo Erba) Flash 2000 Elemental Analyzer, configured for wt % CHN. Thermogravimetric analysis (TGA) was performed in N_2_, on an nSTA 449 F1 Jupiter instrument (heating rate: 10 °C/min; temperature range: 25–800 °C). Powder X-ray diffraction (PXRD) was recorded at room temperature on a Siemens D-5000 diffractometer with Cu Kα radiation (λ = 1.5418 Å, 35 kV, 35 mA, increment = 0.02°). Inductively coupled plasma-mass spectrometry (ICP-MS) measurements were carried out on an Agilent ICP-MS 7700x apparatus. Scanning electron microscopy (SEM) (QUANTA FEI 200 FEGESEM) and optical microscopy (Olympus BX52) were used to monitor the morphology and color changes at various conditions. Solid-state UV–visible spectra were obtained on a UV–Vis–NIR V-780 spectrophotometer equipped with an operational range of 200–1600 nm.

Emission spectra were obtained with a PTI Quantamaster 300 fluorimeter, putting the solid powder in a custom-made holder and setting the holder plane at 45° with the direction of the incident light and the optical path toward the detector. All spectra were obtained on irradiating with a continuous-wave Xe lamp at λ_exc_ = 280 nm. Lifetime measurements were obtained with the same fluorimeter, but at an excitation of 280 nm with a pulsed Xe lamp (100 Hz, 2 μs integration time). Absolute luminescence quantum yields (Φ) of solid-state samples under continuous-wave excitation (λ_ex_ = 280 nm) were determined using the quantum yield fluorimeter Hamamatsu C9920-02G, equipped with an integrating sphere, connected to the lamp with an optical fiber, at room temperature in the air. Φ values were calculated based on the number of photons absorbed and emitted by the sample. A detailed measurement procedure can be found in a previous report.^97^ Reported overall Φ values are averages of at least three independent determinations.

Delay time-dependent emission spectra and bar codes under a pulsed excitation (λ_ex_ = 355 and 266 nm) were recorded irradiating with the fourth and third harmonic of a Nd:YAG (Brilliant B, Spectra Physics) ns pulsed laser. The emission was recorded using an Andor ICCD camera coupled to a spectrograph, setting the sample powder or loaded cellulose papers at 45° with the incident beam and the optical path toward the detector. Measurements were recorded at a 1 Hz frequency, 100 ns (266 nm) or 5000 ns (355 nm) integration time, and applying different delays with respect to the excitation pulse.

#### Computational Details

To analyze the photochemical properties of the ***m*****CB** ligand, computational methods have been employed. The calculations were performed using the Gaussian 16 program^105^ with the TDDFT method and the exchange–correlation functional B3LYP.^106^ Other functionals commonly employed in the TDDFT calculation of organic systems were tested (PBE0,^107^ LC-wPBE^108^). However, due to the larger exact exchange contributions, they provide a more energetic transition than B3LYP and consequently, poorer agreement with the experimental data. The 6-311G* basis set was employed for the geometry optimization and the 6-311+G** basis set for the TDDFT calculations. Neutral molecules including the acidic hydrogen atoms were included because they provide a better description of the metal-coordinated ligands than the anionic ligands.

## Supporting Information Available

Optical and SEM images, IR, TGA, crystallographic data, powder X-ray diffraction, UV–vis spectra, luminescence spectra and decay curves, TTDFT results, and time-dependent emission spectra (PDF)
