# Supplementary material for: Water-Stable Carborane-Based Eu3+/Tb3+ Metal–Organic Frameworks for Tunable Time-Dependent Emission Color and Their Application in Anticounterfeiting Bar-Coding
Source: Chem Mater. 2022 Apr 29;34(10):4795–808. doi: 10.1021/acs.chemmater.2c00323 (PMC9136944; doi:10.1021/acs.chemmater.2c00323)

Supporting information

Water Stable Carborane-based Eu^{3+}/Tb^{3+} Metal Organic Frameworks for Tunable Time-dependent Emission color and its Application in Anticounterfeiting Bar-coding

Zhen Li, Rosario Núñez, Mark E. Light, Eliseo Ruiz, Francesc Teixidor, Clara Viñas, Daniel Ruiz-Molina, Claudio Roscini,* José Giner Planas*

Zhen Li, R. Núñez, F. Teixidor, C. Viñas, J. G. Planas
Institut de Ciència de Materials de Barcelona (ICMAB-CSIC), Campus UAB, 08193 Bellaterra, Spain.
E-mail: jginerplanas@icmab.es
C. Roscini, D. Ruiz-Molina
Catalan Institute of Nanoscience and Nanotechnology (ICN2), CSIC, and The Barcelona Institute of Science and Technology (BIST), Campus UAB, Bellaterra, Barcelona 08193, Spain.
E-mail: claudio.roscini@icn2.cat
M. E. Light
Department of Chemistry, University of Southampton, Highfield, Southampton SO17 1BJ, UK.
E. Ruiz
Departament de Química Inorgànica i Orgànica and Institut de Recerca de Química Teòrica i Computacional, Universitat de Barcelona, Diagonal 645, 08028 Barcelona, Spain.

Supplementary Figures and Tables

Figure S1. Optical images of the crystals of ***m*CB-Tb** (a), ***m*CB-Eu** (b) and ***m*CB-Eu$_{0.10}$Tb$_{0.90}$** (c).

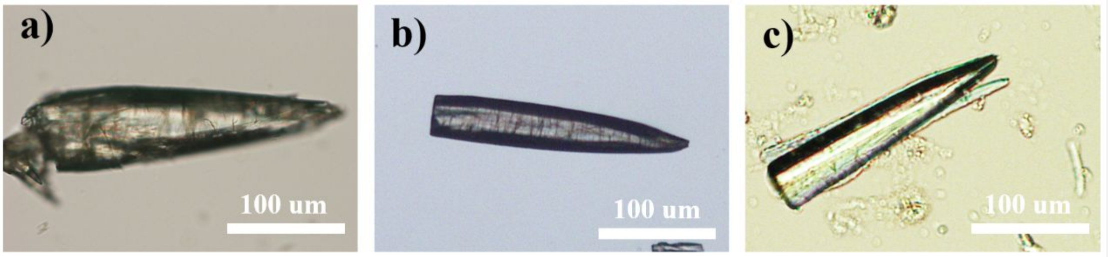

Figure S2. FTIR spectra for ***m*CB-Tb** (black), ***m*CB-Eu** (red) and ***m*CB-Eu$_{0.10}$Tb$_{0.90}$**.

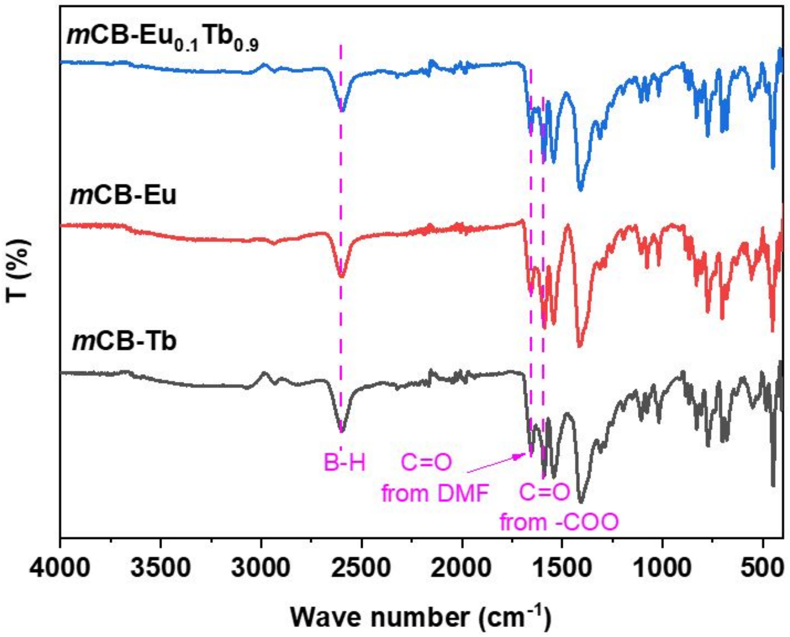

Crystallography.

**Table S1**. Crystal and Structure Refinement data for ***m*CB-Tb**.	
Compound	***m*CB-Tb**
Empirical formula	$C_{76}H_{100}B_{40}N_5O_{23}Tb_3$
Formula weight	2360.76
Crystal system	Monoclinic
Space group	*Pn*
CCDC ref	2101866
Wavelength (Å)	0.71073
Temperature	100(2)K
a (Å)	12.0715(2)
b (Å)	13.5823(2)
c (Å)	32.3709(4)
β (°)	90.0940(10)
V (Å^3)	5307.48(13)
Z	2
$\rho_{(calc)}$ (g/cm^3)	1.477
F (000)	2340
θ range (deg)	3.442- 28.500
Max./min. transmission	1.000 / 0.666
Ind refln<br>(R_{int})	24410<br>0.0594
Goodness-of-fit on F^2	1.047
R_1 [a] ($I > 2\sigma(I)$)<br>R_1 [a] (all data)<br>wR_2 [b] ($I > 2\sigma(I)$)<br>wR_2 [b] (all data)	0.0503<br>0.0576<br>0.1303<br>0.1355

[a] $R_1 = \Sigma(||F_0|-|F_C||)/\Sigma|F_0|$.
[b] $wR_2 = [\Sigma w(|F_0|^2-|F_C|^2)^2/\Sigma w(F_0^2)]^{1/2}$.

Figure S3. PXRD patterns of: (a) experimental ***m*CB-Tb** and ***m*CB-Eu** and simulated ***m*CB-Tb**; (b) experimental mixed ***m*CB-Eu$_y$Tb$_{1-y}$**.

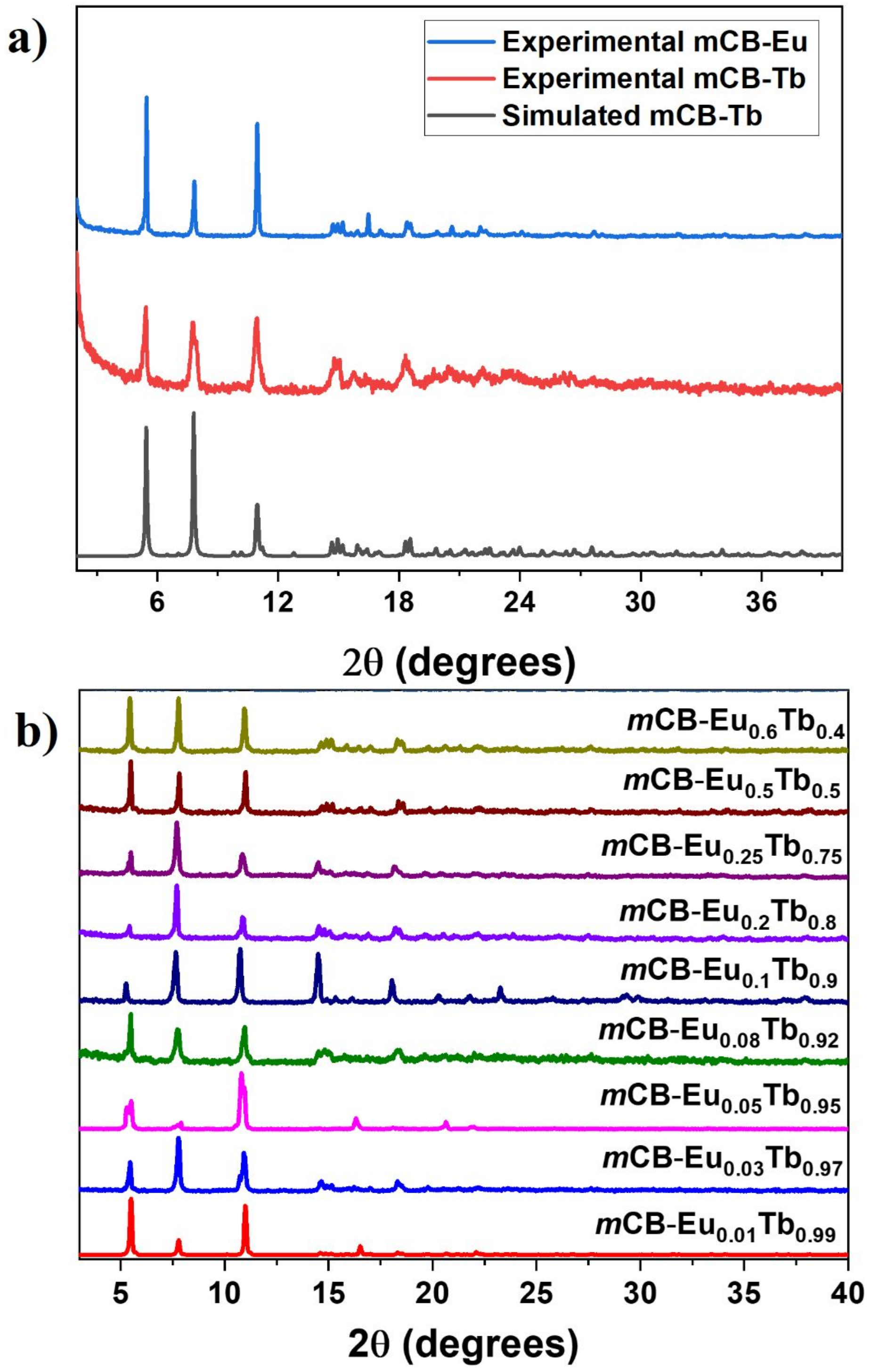

Figure S4. TGA curves for ***m*CB-Tb** (a) and ***m*CB-Eu** (b).

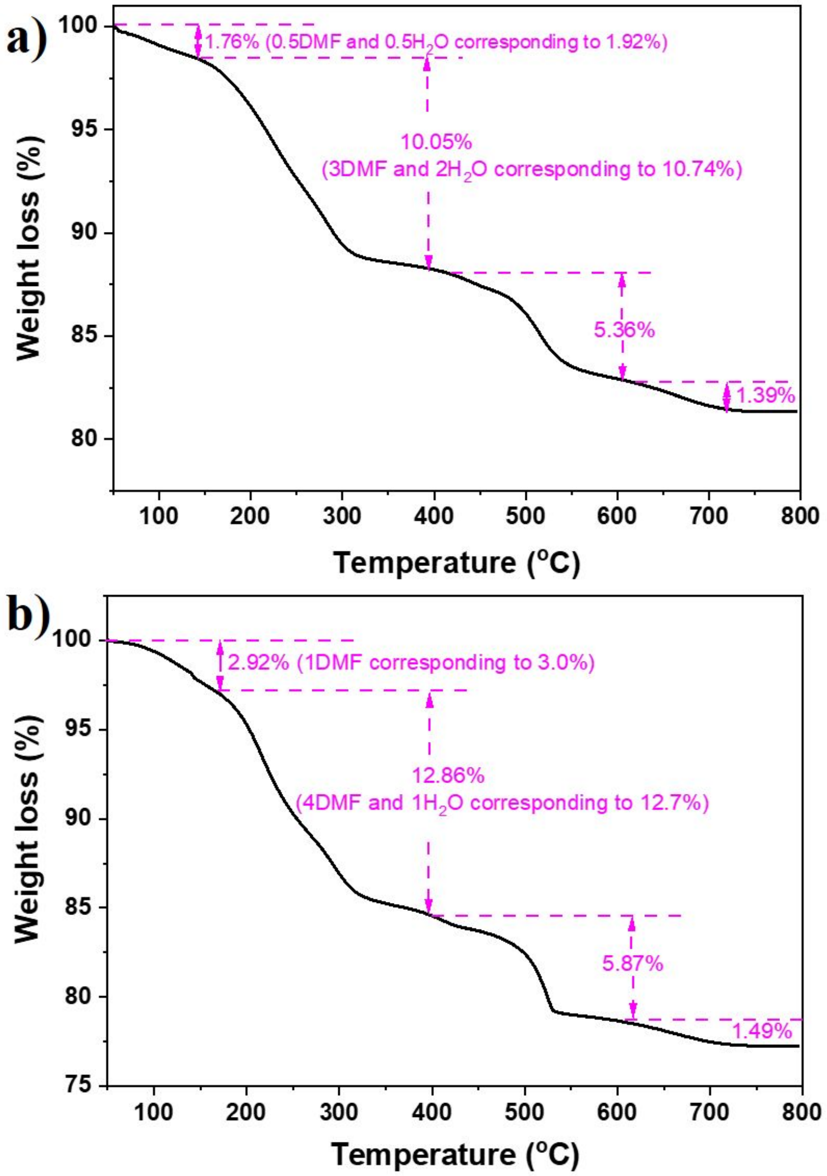

Figure S5. PXRD patterns (left) and optical images (right) under visible and UV light of the corresponding crystals for ***m*CB-Tb** (a) and ***m*CB-Eu** (b) at various conditions.

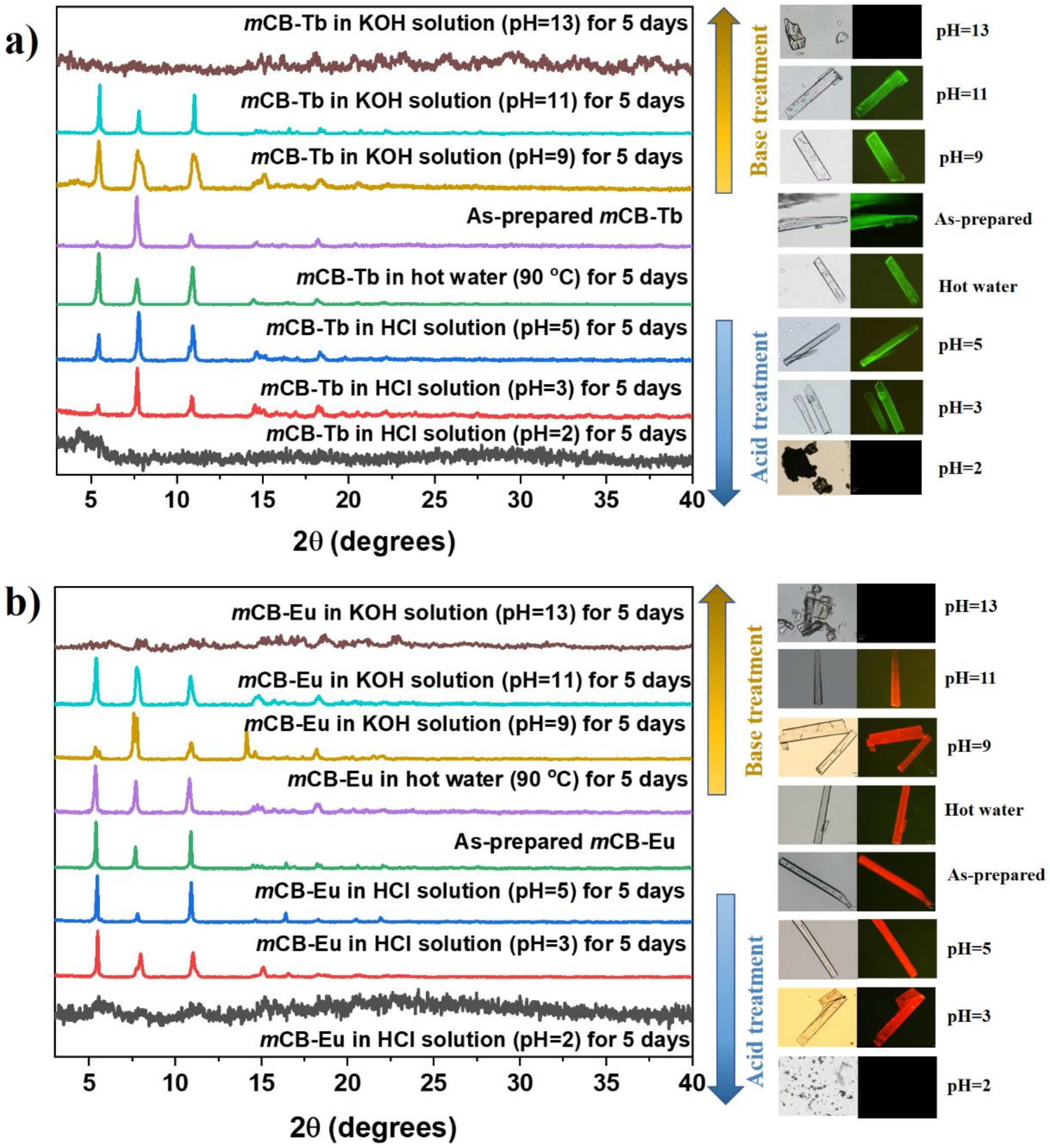

Figure S6. UV-vis spectra for solid *m*CBL1 and the corresponding ***m*CB-Ln** samples.

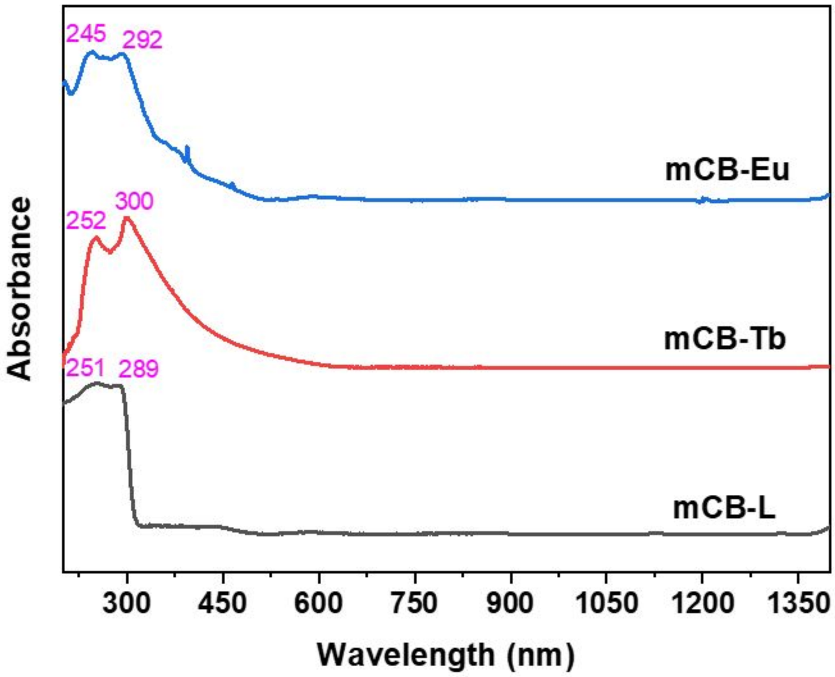

Figure S7. Luminescence spectra for *m*CBL1 ligand under continuous excitation at 280 nm.

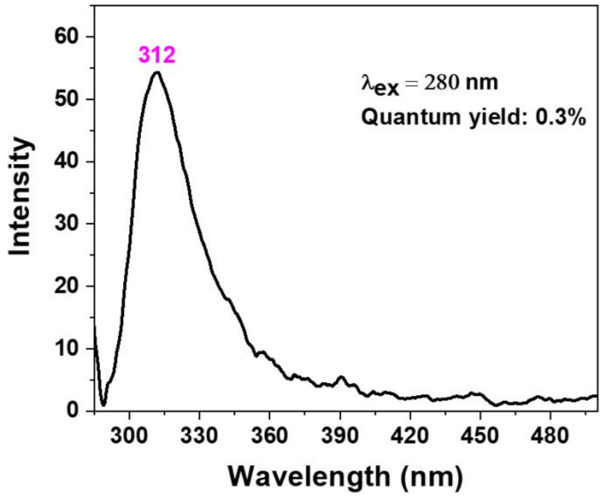

Figure S8. Optical images under white light (left) or UV-light (right) for ***m*CB-Tb** (top) and ***m*CB-Eu** (bottom) crystals after heating treatment at 180 ^{o}C.

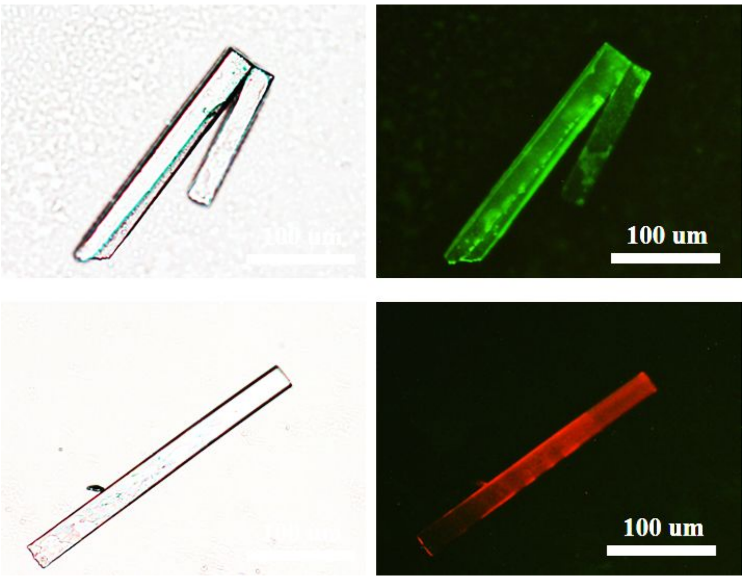

Figure S9. SEM images of a sample MOF suspension deposited on paper.

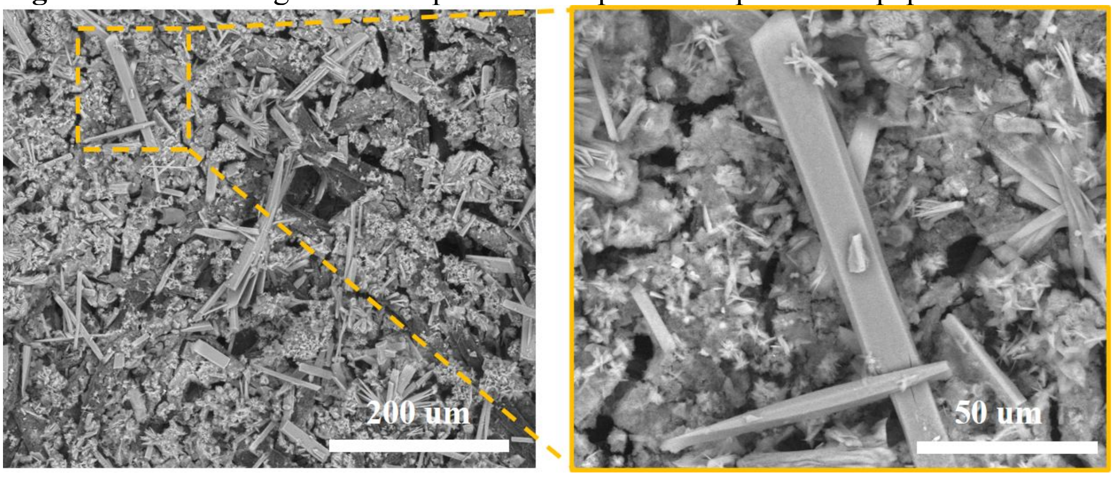

Figure S10. Emission spectra of ***m*CB-Eu** (a) and ***m*CB-Tb** (b) before and after deposited on the paper under continuous wave irradiation (λ_{ex} = 280 nm) at room temperature.

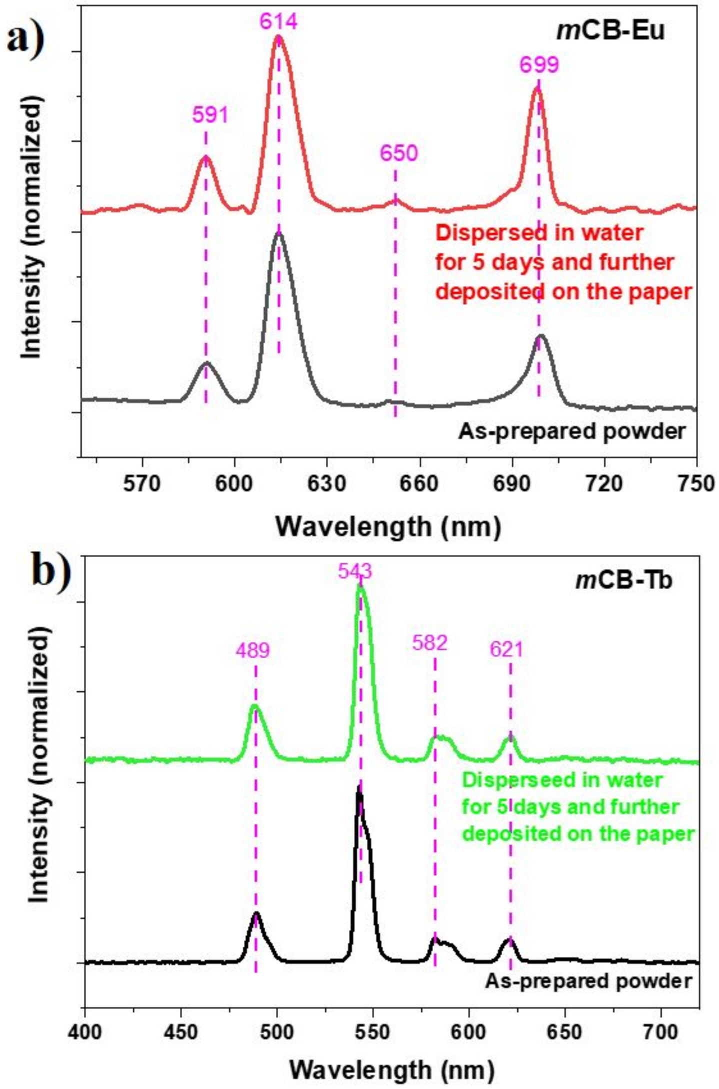

Figure S11. The orbitals involved in the first singlet excitation and the triplet emission of the *m*TDCA ligand.

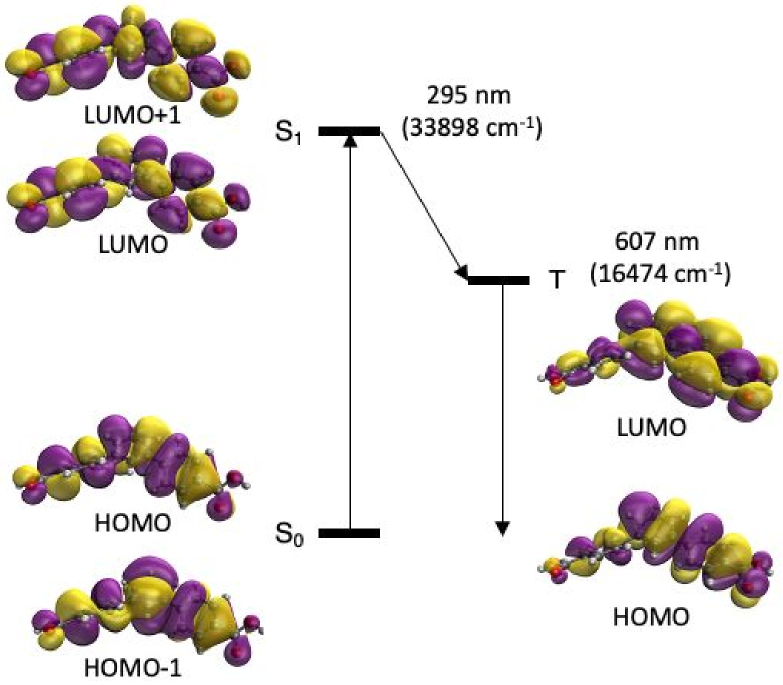

Figure S12. B3LYP optimized structures for the ground state singlet (**S_0**) and for the first triplet (**T**) state of the *m*CBL1 and *m*TDCA ligands.

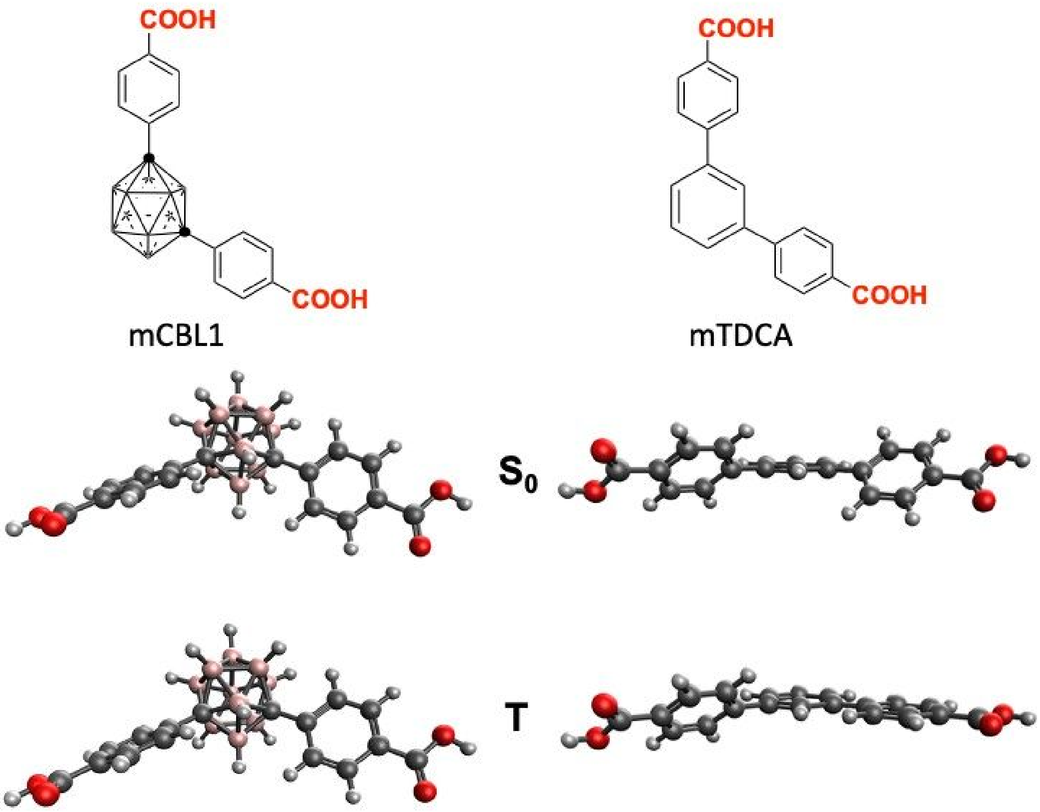

Figure S13. Schematic diagram of the energy absorption to the singlet states (S_0) of *m*CBL1 (left) and *m*TDCA (right) ligands, transfer to the triplet states (T_1) and emission processes of Eu- and Tb-MOFs. Dashed arrows represent an expected less efficient transfer.

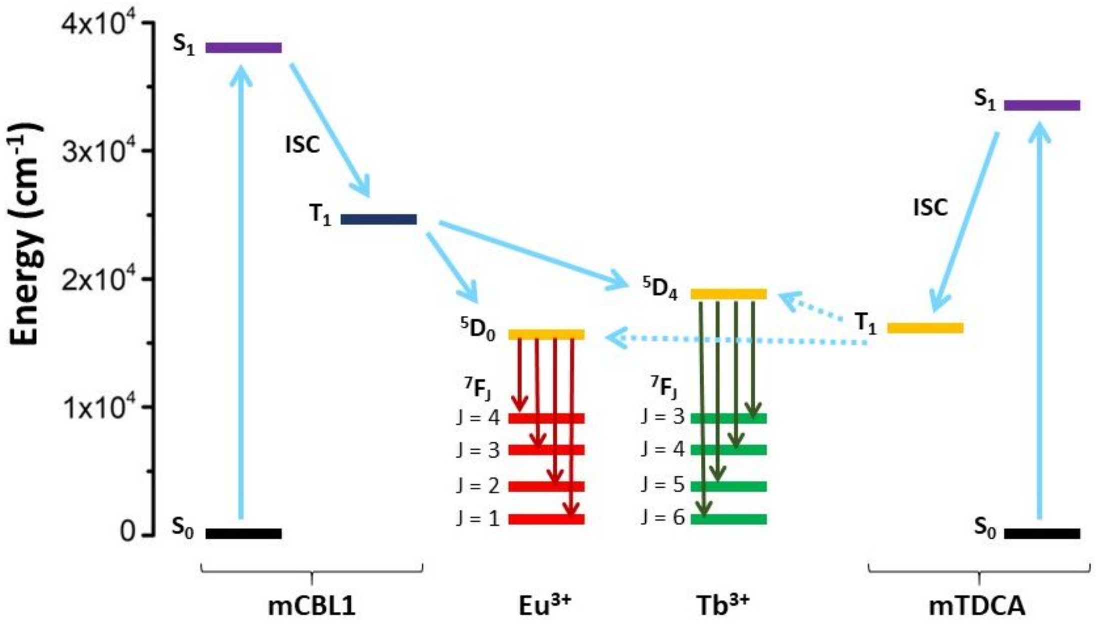

Table S2. Summary of ICP results and the corresponding Eu/Tb ratios in the mixed ***m*CB-Eu$_y$Tb$_{1-y}$** samples.

Sample Name	ICP results		Experimental Eu/Tb molar ratios
	Eu	Tb	
***m*CB-Eu$_{0.60}$Tb$_{0.40}$**	10.8	6.8	0.62/0.38
***m*CB-Eu$_{0.50}$Tb$_{0.50}$**	8.3	8.6	0.50/0.50
***m*CB-Eu$_{0.25}$Tb$_{0.75}$**	6.5	12.7	0.35/0.65
***m*CB-Eu$_{0.20}$Tb$_{0.80}$**	3.8	13.9	0.22/0.78
***m*CB-Eu$_{0.10}$Tb$_{0.90}$**	1.8	17.9	0.10/0.90
***m*CB-Eu$_{0.08}$Tb$_{0.92}$**	1.5	15.4	0.09/0.91
***m*CB-Eu$_{0.05}$Tb$_{0.95}$**	1.2	16	0.07/0.93
***m*CB-Eu$_{0.03}$Tb$_{0.97}$**	0.8	16.7	0.05/0.95
***m*CB-Eu$_{0.01}$Tb$_{0.99}$**	0.3	16.6	0.02/0.98

Figure S14. Solid-state emission spectra of mixed ***m*****CB-Eu$_y$Tb$_{1-y}$** with various Eu/Tb molar ratios under a steady excitation.

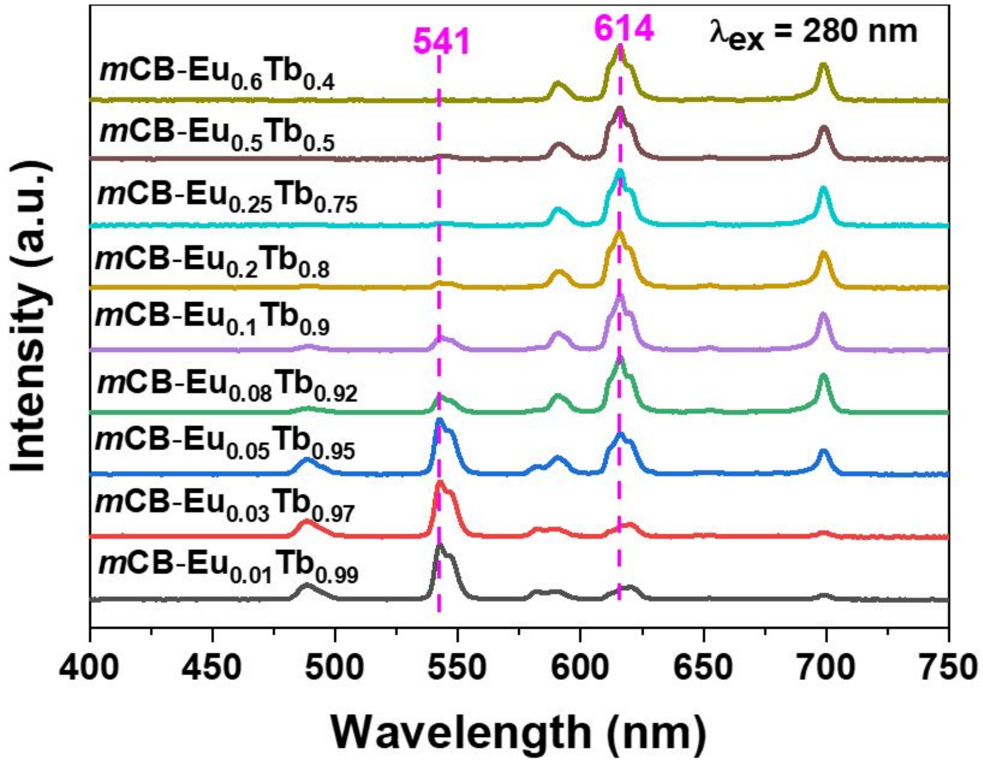

Figure S15 Luminescence decay curves for (a) ***m*CB-Tb** (λ_{ex} = 280 nm, λ_{em} = 541 nm) and (b) ***m*CB-Eu** (λ_{ex} = 280 nm, λ_{em} = 614 nm) samples at RT and the corresponding fitting curves with a R^2 value of 0.98 for both ***m*CB-Tb and *m*CB-Eu**.

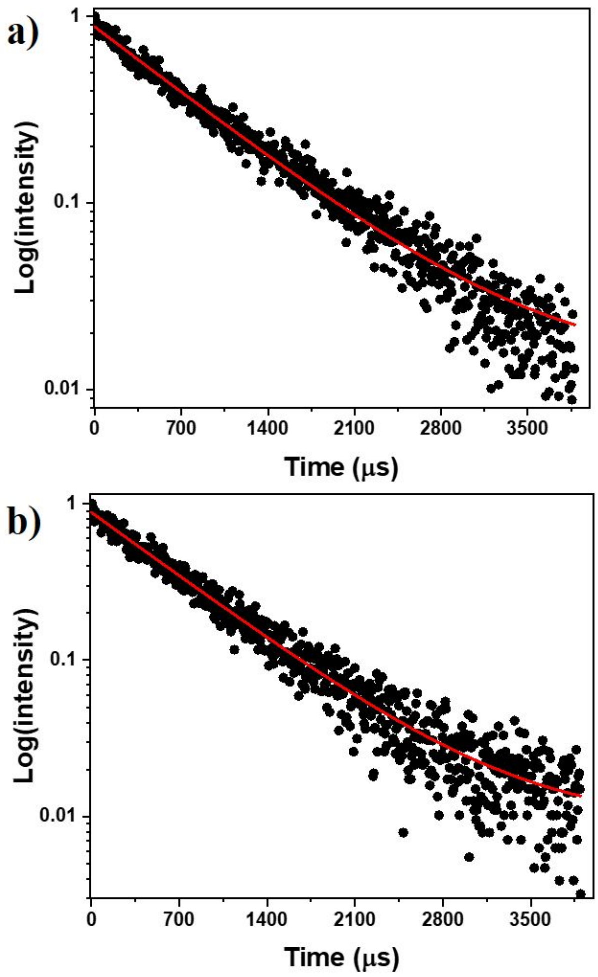

Figure S16: fluorescence decays of Tb (λ_{em} = 541 nm) in the different MOFs (λ_{exc} = 280 nm).

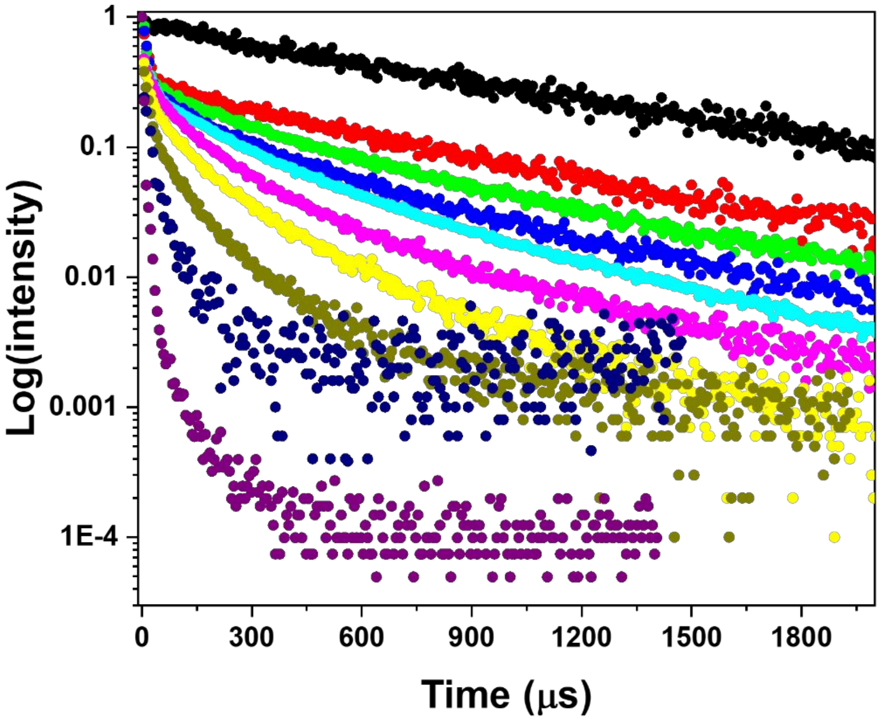

Figure S17. fluorescence decays of Eu (λ_{em} = 614 nm) in the different MOFs (λ_{exc} = 280 nm).

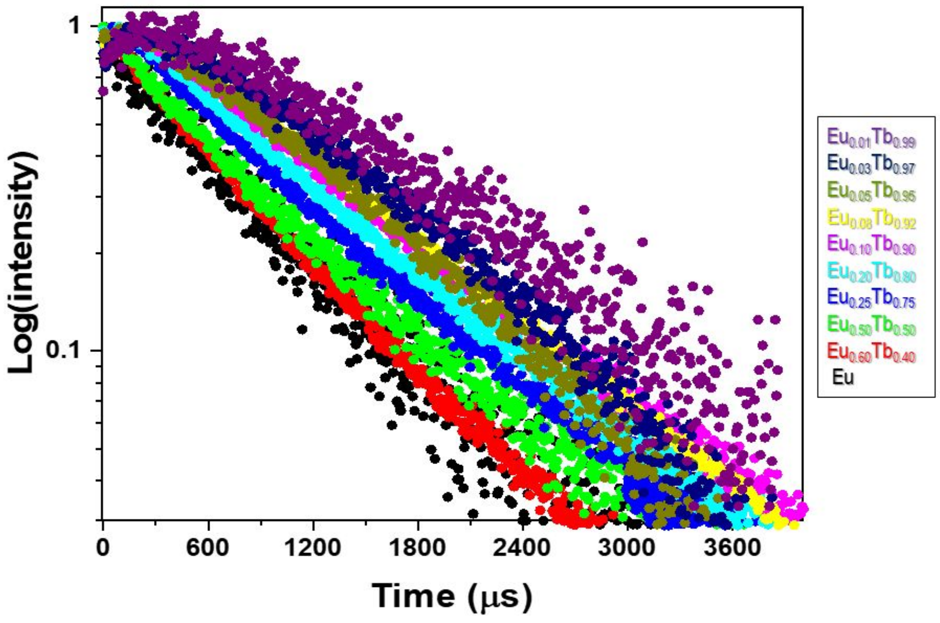

Figure S18. Luminescence decay curves for (a) Tb^{3+} (λ_{ex} = 280 nm, λ_{em} = 541 nm) and (b) Eu^{3+} (λ_{ex} = 280 nm, λ_{em} = 614 nm) ions in ***m*CB-Eu$_{0.01}$Tb$_{0.99}$** at RT and the corresponding fitting curves with a R^2 value of 0.99 and 0.96 for Tb^{3+} and Eu^{3+}, respectively.

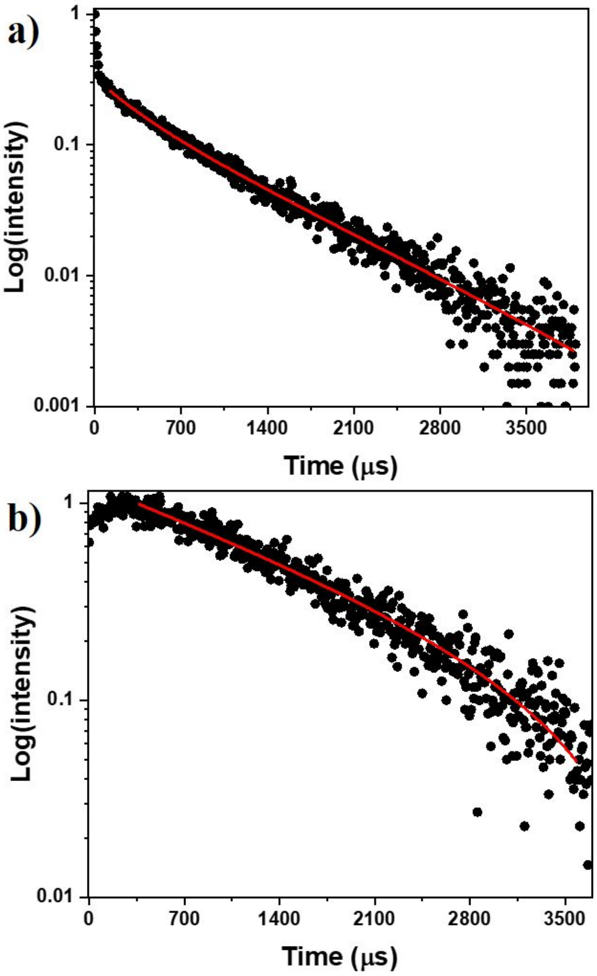

Figure S19. Luminescence decay curves for (a) Tb^{3+} (λ_{ex} = 280 nm, λ_{em} = 541 nm) and (b) Eu^{3+} (λ_{ex} = 280 nm, λ_{em} = 614 nm) ions in ***m*CB-Eu$_{0.03}$Tb$_{0.97}$** at RT and the corresponding fitting curves with a R^2 value of 0.99 for both Tb^{3+} and Eu^{3+}.

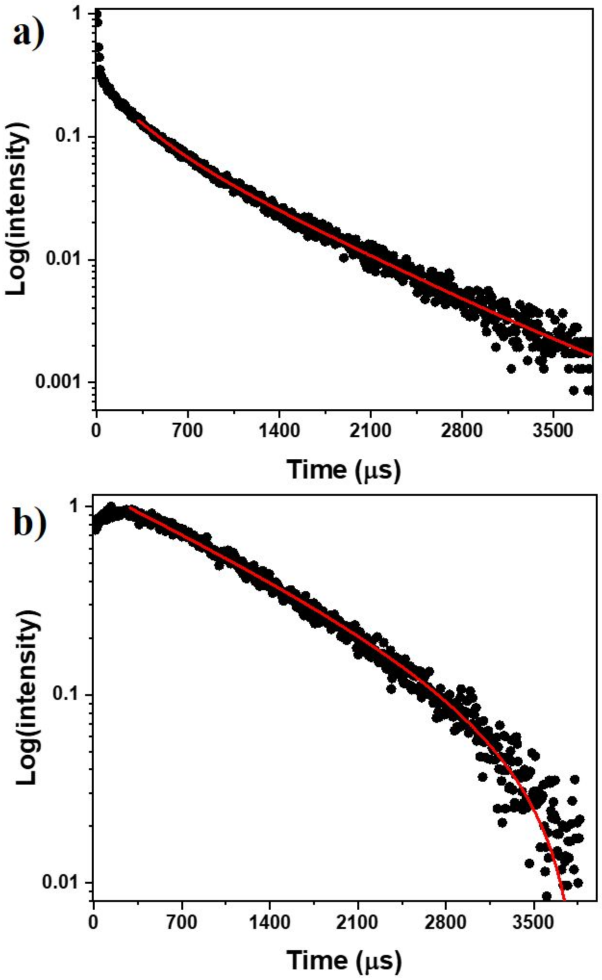

Figure S20. Luminescence decay curves for (a) Tb^{3+} (λ_{ex} = 280 nm, λ_{em} = 541 nm) and (b) Eu^{3+} (λ_{ex} = 280 nm, λ_{em} = 614 nm) ions in ***m*****CB-Eu$_{0.05}$Tb$_{0.95}$** at RT and the corresponding fitting curves with a R^2 value of 0.99 for both Tb^{3+} and Eu^{3+}.

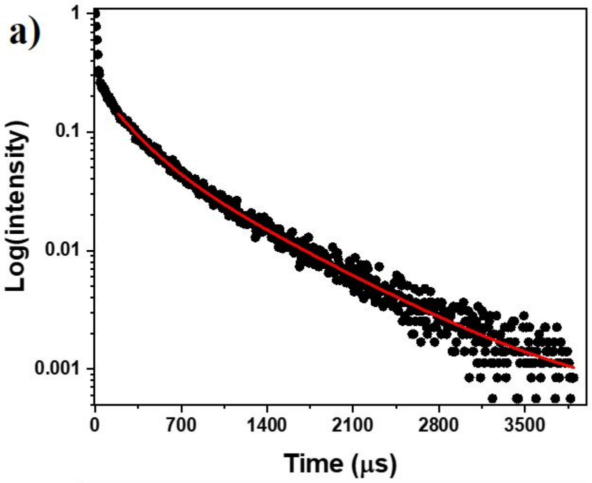

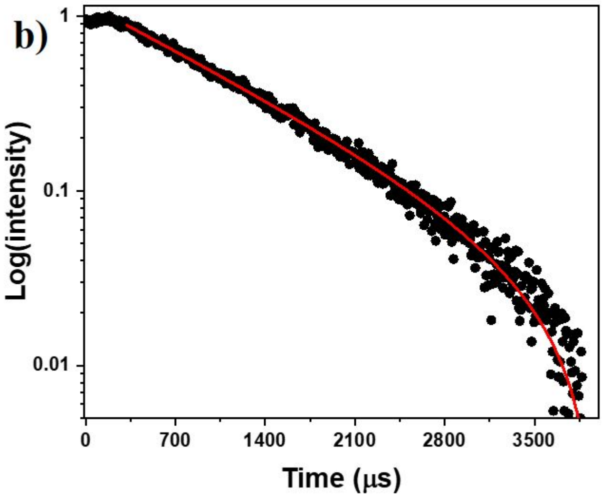

Figure S21. Luminescence decay curves for (a) Tb^{3+} (λ_{ex} = 280 nm, λ_{em} = 541 nm) and (b) Eu^{3+} (λ_{ex} = 280 nm, λ_{em} = 614 nm) ions in ***m*CB-Eu$_{0.08}$Tb$_{0.92}$** at RT and the corresponding fitting curves with a R^2 value of 0.99 for both Tb^{3+} and Eu^{3+}.

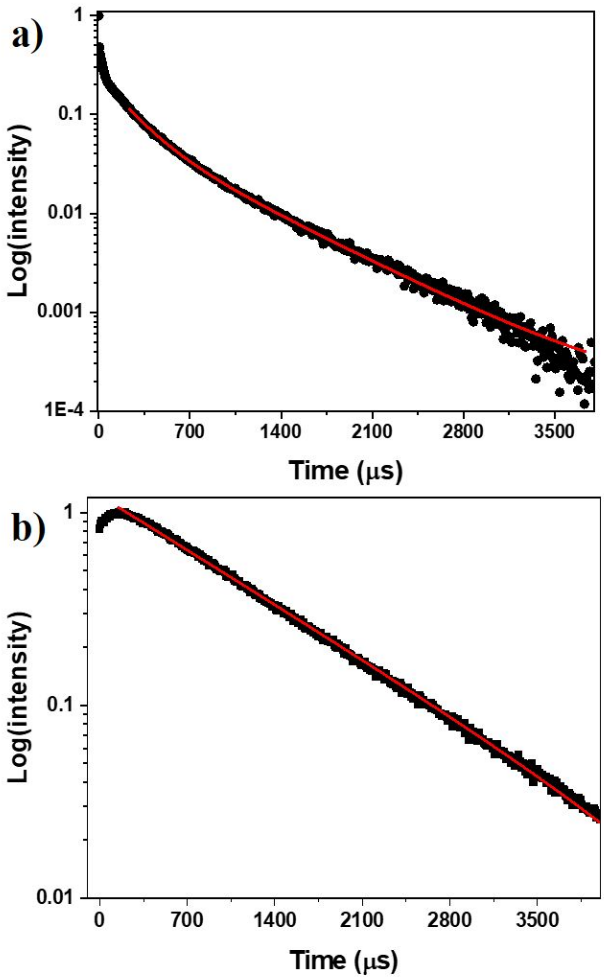

Figure S22. Luminescence decay curves for (a) Tb^{3+} (λ_{ex} = 280 nm, λ_{em} = 541 nm) and (b) Eu^{3+} (λ_{ex} = 280 nm, λ_{em} = 614 nm) ions in ***m*****CB-Eu$_{0.10}$Tb$_{0.90}$** at RT and the corresponding fitting curves with a R^2 value of 0.98 for both Tb^{3+} and Eu^{3+}.

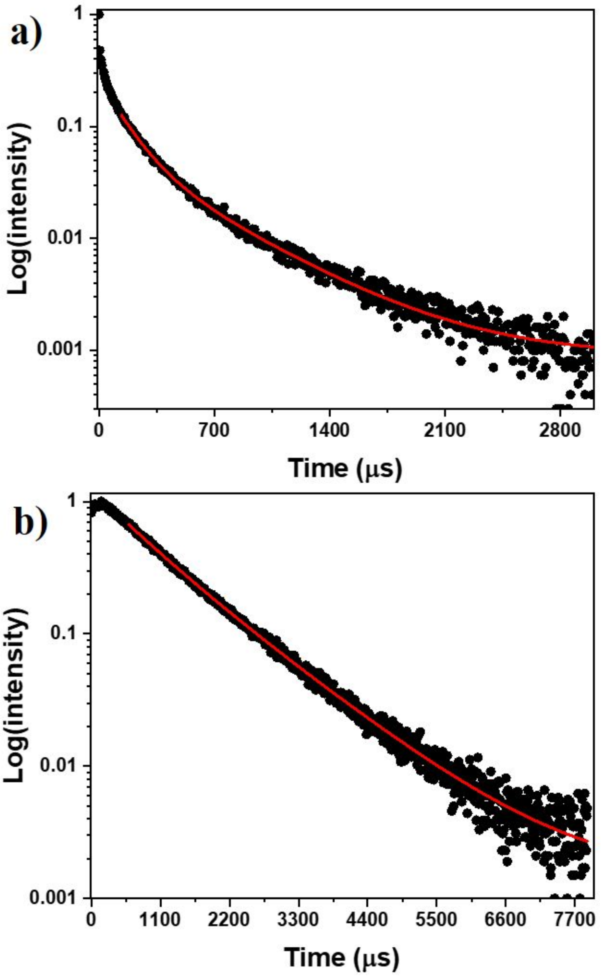

Figure S23. Luminescence decay curves for (a) Tb^{3+} (λ_{ex} = 280 nm, λ_{em} = 541 nm) and (b) Eu^{3+} (λ_{ex} = 280 nm, λ_{em} = 614 nm) ions in ***m*****CB-Eu$_{0.20}$Tb$_{0.80}$** at RT and the corresponding fitting curves with a R^2 value of 0.99 for both Tb^{3+} and Eu^{3+}.

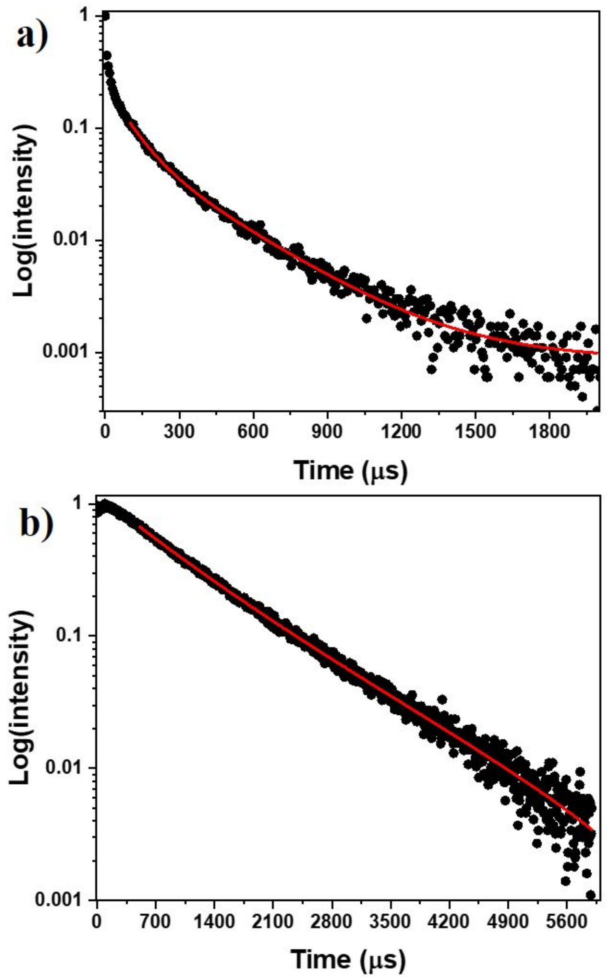

Figure S24. Luminescence decay curves for (a) Tb^{3+} (λ_{ex} = 280 nm, λ_{em} = 541 nm) and (b) Eu^{3+} (λ_{ex} = 280 nm, λ_{em} = 614 nm) ions in ***m*CB-Eu$_{0.25}$Tb$_{0.75}$** at RT and the corresponding fitting curves with a R^2 value of 0.99 for both Tb^{3+} and Eu^{3+}.

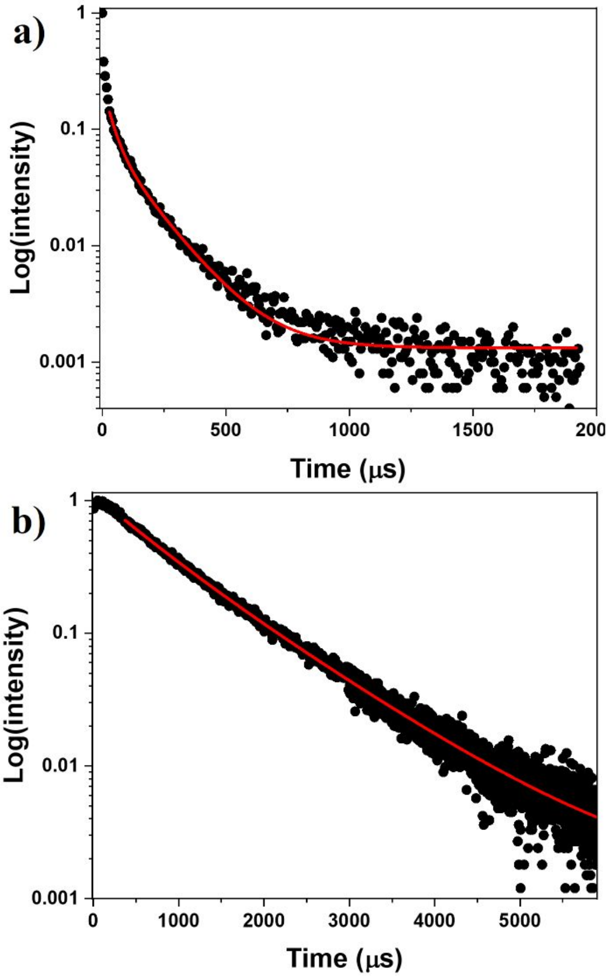

Figure S25. Luminescence decay curves for (a) Tb^{3+} (λ_{ex} = 280 nm, λ_{em} = 541 nm) and (b) Eu^{3+} (λ_{ex} = 280 nm, λ_{em} = 614 nm) ions in ***m*CB-Eu$_{0.50}$Tb$_{0.50}$** at RT and the corresponding fitting curves with a R^2 value of 0.99 for both Tb^{3+} and Eu^{3+}.

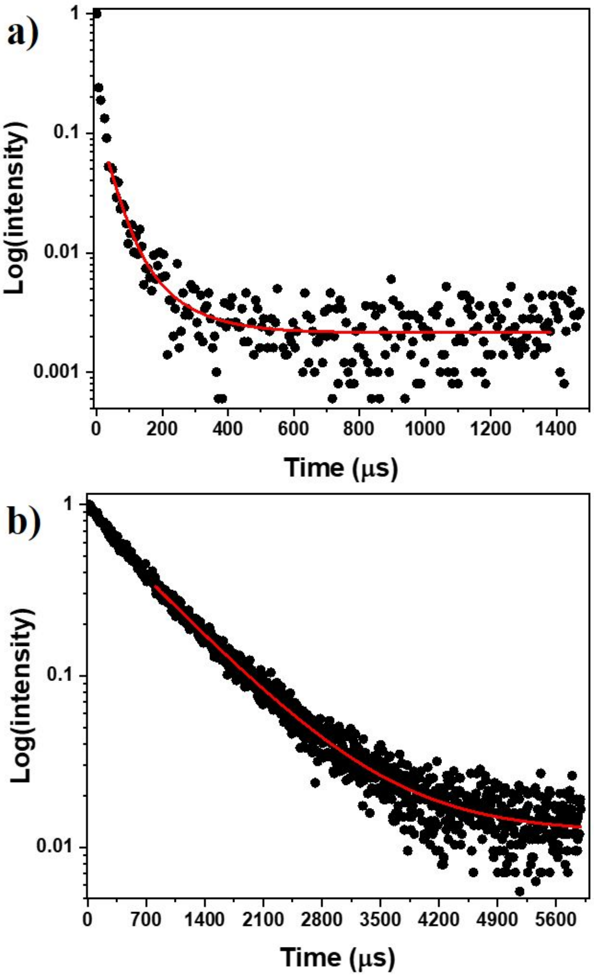

Figure S26. Luminescence decay curves for (a) Tb^{3+} (λ_{ex} = 280 nm, λ_{em} = 541 nm) and (b) Eu^{3+} (λ_{ex} = 280 nm, λ_{em} = 614 nm) ions in ***m*CB-Eu$_{0.60}$Tb$_{0.40}$** at RT and the corresponding fitting curves with a R^2 value of 0.99 for both Tb^{3+} and Eu^{3+}.

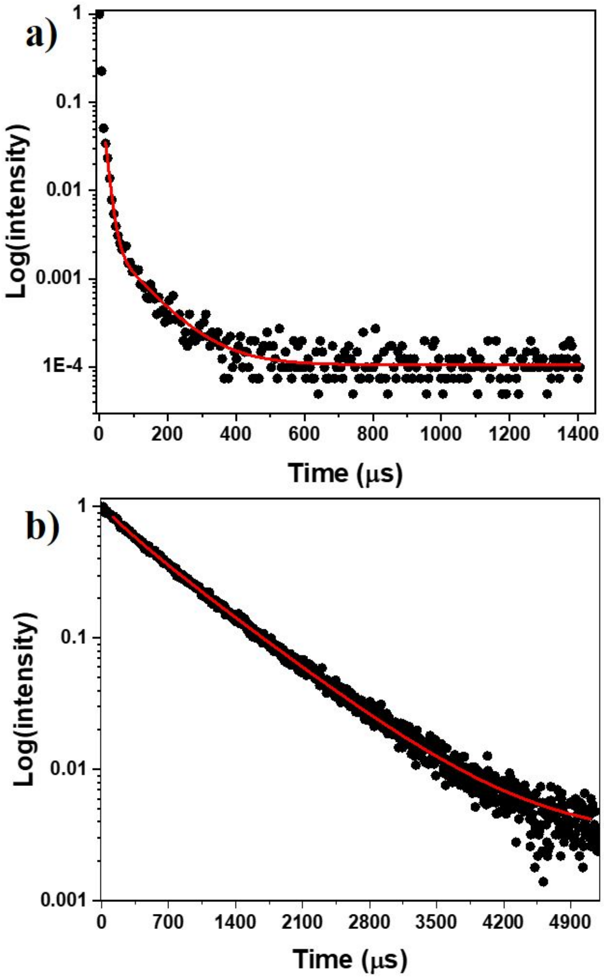

Figure S27. Time-dependent emission spectra and the corresponding time-dependent bar codes of ***m*CB-Eu$_{0.01}$Tb$_{0.99}$** powder (λ_{ex} = 266 nm).

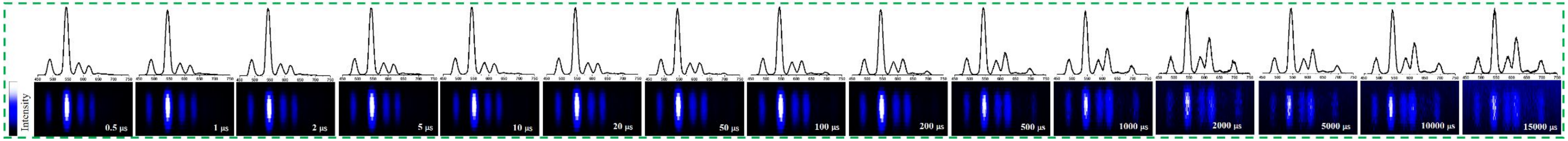

Figure S28. Time-dependent emission spectra and the corresponding time-dependent bar codes of ***m*CB-Eu$_{0.1}$Tb$_{0.9}$** powder (λ_{ex} = 266 nm).

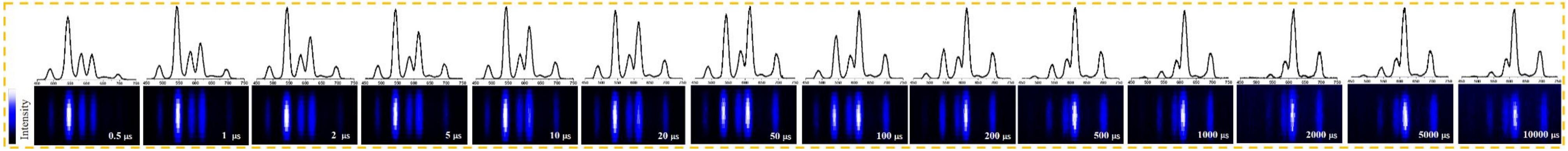

Figure S29. Time-dependent emission spectra and the corresponding time-dependent bar codes of ***m*CB-Eu$_{0.6}$Tb$_{0.4}$** powder (λ_{ex} = 266 nm).

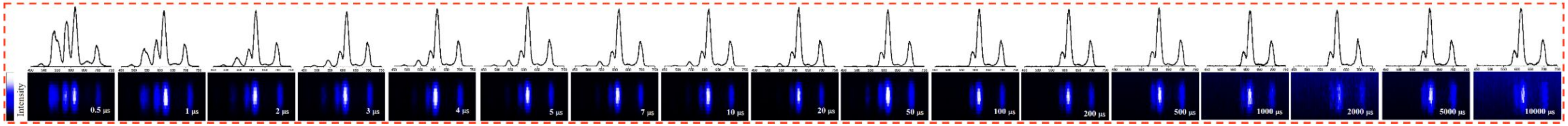

Figure S30. Time-dependent emission spectra and the corresponding time-dependent bar codes of ***m*CB-Eu$_{0.1}$Tb$_{0.9}$** powder (λ_{ex} = 355 nm).

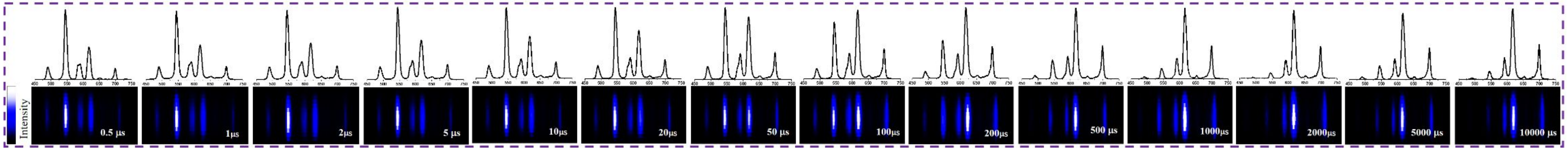

Figure S31. Time-dependent emission spectra and the corresponding time-dependent bar codes of ***m*CB-Eu$_{0.1}$Tb$_{0.9}$** deposited on the paper (λ_{ex} = 266 nm).

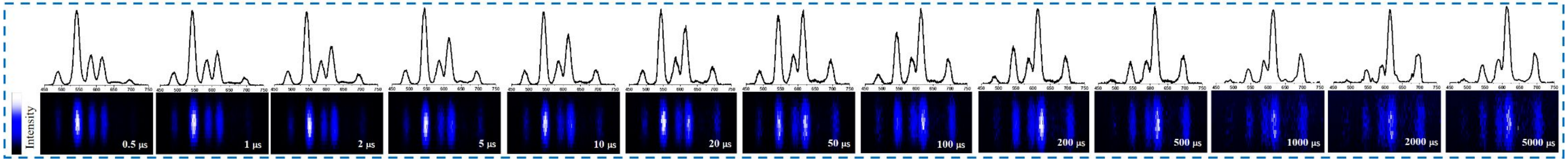

Figure S32: **a**) Time-dependent emission spectra of the printed ***m*CB-Eu$_{0.01}$Tb$_{0.99}$** and b) corresponding color coordinates in the 1931 CIE diagram; *c)* Time-dependent bar codes of the printed ***m*CB-Eu$_{0.01}$Tb$_{0.99}$** (λ_{exc} = 355 nm).

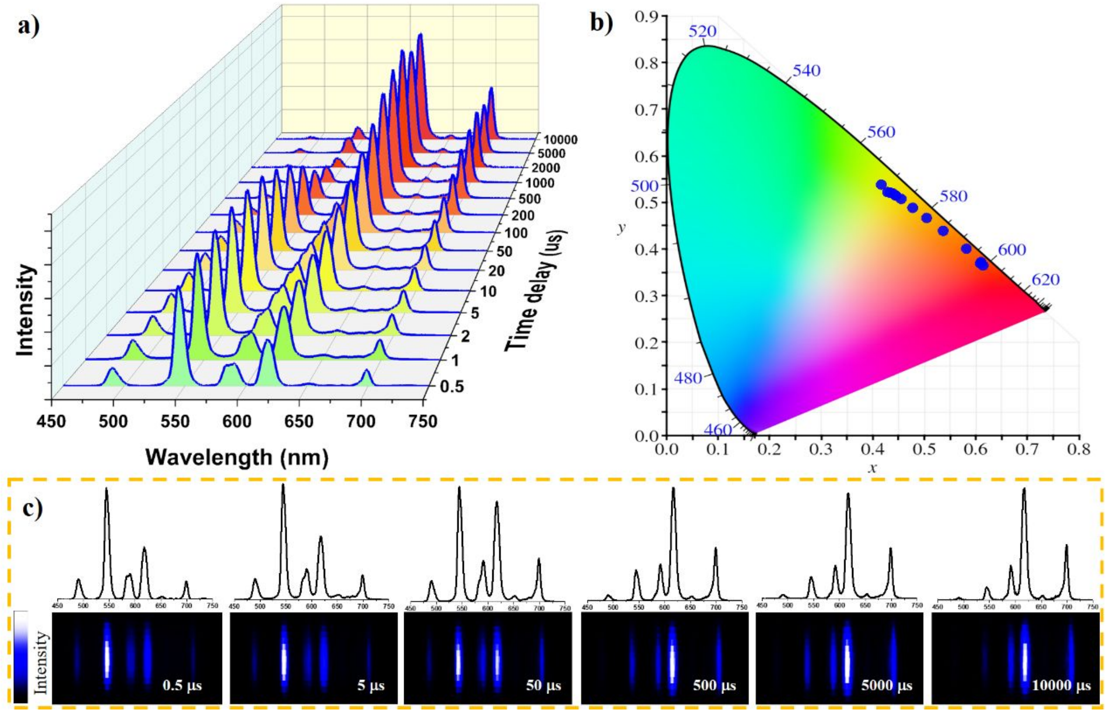

Figure S33: scheme of spray-coating technique adapted to print the ***m*CB-Eu$_{0.1}$Tb$_{0.9}$** particles.

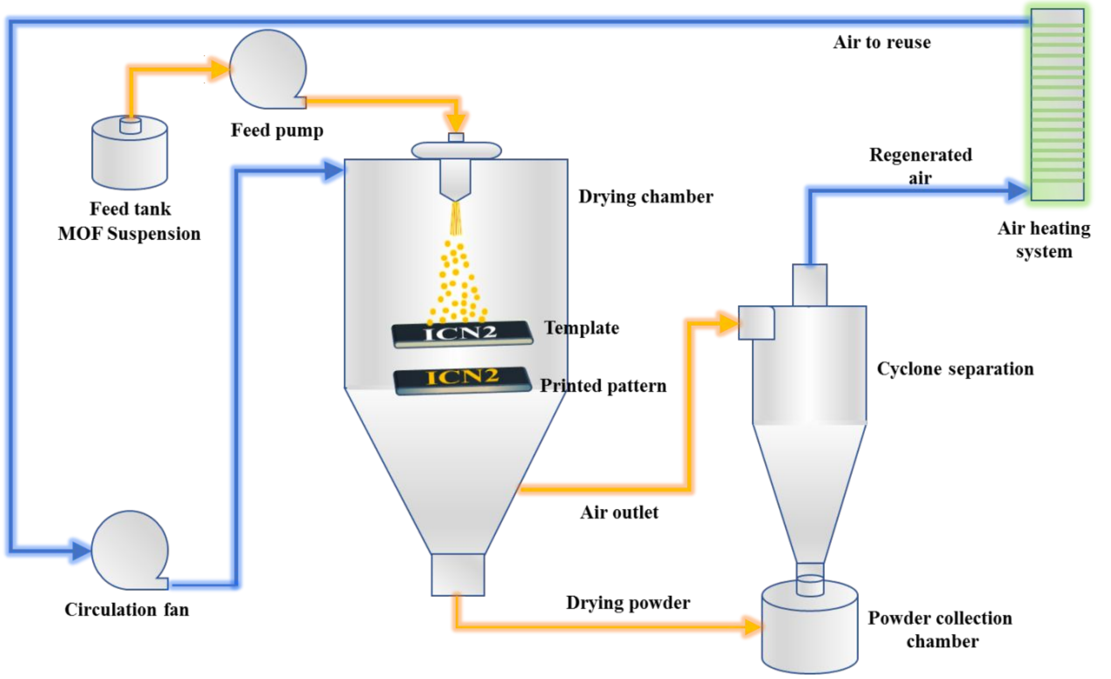

Figure S34. Emission spectra for ***m*CB-Eu$_{0.10}$Tb$_{0.90}$** deposited on the paper under steady excitation (280 nm). (Insert: luminescent photograph of the ***m*CB-Eu$_{0.10}$Tb$_{0.90}$** aqueous ink)

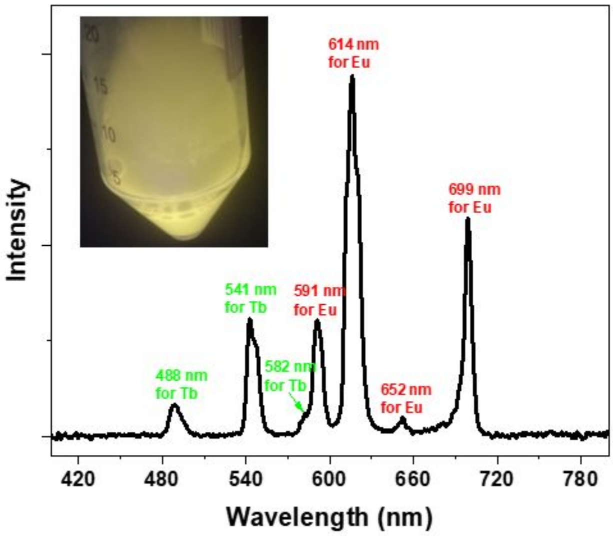

Supplement: Supplementary file 1 — cm2c00323_si_001.pdf [file cm2c00323_si_001.pdf]
